# *Mef2d* Acts Upstream of Muscle Identity Genes and Couples Lateral Myogenesis to Dermomyotome Formation in *Xenopus laevis*

**DOI:** 10.1371/journal.pone.0052359

**Published:** 2012-12-31

**Authors:** Bruno Della Gaspera, Anne-Sophie Armand, Sylvie Lecolle, Frédéric Charbonnier, Christophe Chanoine

**Affiliations:** Centre d’Etude de la Sensori-Motricité, UMR 8194 CNRS, Université Paris Descartes, Centre Universitaire des Saints-Pères, Paris, France; University of Minnesota Medical School, United States of America

## Abstract

*Xenopus* myotome is formed by a first medial and lateral myogenesis directly arising from the presomitic mesoderm followed by a second myogenic wave emanating from the dermomyotome. Here, by a series of gain and loss of function experiments, we showed that *Mef2d*, a member of the Mef2 family of MADS-box transcription factors, appeared as an upstream regulator of lateral myogenesis, and as an inducer of dermomyotome formation at the beginning of neurulation. In the lateral presomitic cells, we showed that *Mef2d* transactivates *Myod* expression which is necessary for lateral myogenesis. In the most lateral cells of the presomitic mesoderm, we showed that *Mef2d* and *Paraxis (Tcf15)*, a member of the Twist family of transcription factors, were co-localized and activate directly the expression of *Meox2*, which acts upstream of *Pax3* expression during dermomyotome formation. Cell tracing experiments confirm that the most lateral *Meox2* expressing cells of the presomitic mesoderm correspond to the dermomyotome progenitors since they give rise to the most dorsal cells of the somitic mesoderm. Thus, *Xenopus Mef2d* couples lateral myogenesis to dermomyotome formation before somite segmentation. These results together with our previous works reveal striking similarities between dermomyotome and tendon formation in *Xenopus*: both develop in association with myogenic cells and both involve a gene transactivation pathway where one member of the Mef2 family, *Mef2d* or *Mef2c*, cooperates with a bHLH protein of the Twist family, *Paraxis* or *Scx* (*Scleraxis*) respectively. We propose that these shared characteristics in *Xenopus laevis* reflect the existence of a vertebrate ancestral mechanism which has coupled the development of the myogenic cells to the formation of associated tissues during somite compartmentalization.

## Introduction

Embryonic and foetal muscle fiber development in vertebrates takes place by the appearance of successive myogenic waves. However, myotome formation presents some differences between mammals and anamniotes. In mammals, newly formed somites are a naive tissue which subdivides into dorsal dermomyotome and ventral sclerotome [1]. Next myogenic cells arising from dermomyotomal lips give rise to myotome. So, dermomyotome formation is the initial event of myogenesis and all muscle cells of the trunk and limbs derive from the dermomyotome [2]. In anamiotes, somites are not a naive tissue since the first myogenesis leads to early myotome formation [3]–[6]. The initial subdivision of the myotome between medial and lateral myogenic populations appears as a common scheme in anammiote myogenesis [3]–[6]. In *Xenopus*, medial and lateral myogenesis develops directly from presomitic mesoderm. During neurulation, the medial myogenic cells differentiate first before somite formation and gives rise to muscle fibers located near the notochord. Next, the lateral myogenic cells differentiate at the time of somitogenesis and gives rise to dorsomedial and ventrolateral cell populations [6]. The second myogenic wave which takes place at the tailbud stage arises from epaxial and hypaxial levels of the demomyotome [7], [8] like in amniotes. The *Xenopus* dermomyotome has been indeed described at the tailbud stage and is constituted by a cell layer on the dorsal surface of the somites [8]. However, the dermomyotome formation in *Xenopus*, probably takes place earlier in a particular context where presomitic cells are already subjected to myogenic signals.

Myogenesis is regulated by the four basic helix-loop-helix transcription factors, Myod, Myf5, Mrf4 (Myf6) and Myogenin (Mgn), known as the muscle regulatory factors (MRFs). Genetic circuitry regulating myogenesis has evolved depending on the location in the body, either through the myogenic program itself, or through the upstream regulators of the determination factors [9]. In zebrafish and mouse, *Myf5* and *Myod* act as determination factors during myogenesis. *Mrf4* has also been identified as a determination factor but only in mouse somites during embryonic myogenesis in *Myf5*/*Myod* double mutant [2], [10]. In the same way, absence of *Myf5* and *Mrf4* affects specifically myogenesis in extraocular muscles during mouse craniofacial development [11]. Moreover, *Myf5*-dependent and -independent lineage was revealed by ablation of *Myf5*-expressing cells during mouse somitogenesis [12], [13]. Distinct myogenic programs expressing only *Myf5*, only *Myod* or both have also been characterized during somitic and craniofacial myogenesis in *Xenopus* [6]. Concerning the upstream regulators of myogenesis, *Pitx2* and *Tbx1* play a major role in activating myogenesis in craniofacial muscles [11], while *Pax3* and *Pax7* are expressed in myogenic precursors in mouse somites [9].

In this work, we focused on the molecular determinants specifying the lateral presomitic cells to a myogenic fate and we identified *Mef2d* as an upstream regulator of muscle identity genes. The myocyte enhancer factor 2 (Mef2) family of MADS (MCMI, agamous, deficiens, serum response factor) box transcription factors has four members in vertebrates, Mef2A, -B, -C and -D. Mef2 proteins form homo- and hetero-dimers and bind to a conserved A/T-rich sequence known as a MEF2 site. Mef2 acts through protein–protein interactions with other transcription factors, to either activate or inhibit specific sets of target genes. The Mef2 family proteins bind directly promoters or enhancers of the majority of muscle genes and interact with members of the *Myod* family of basic helix–loop–helix (bHLH) proteins to activate the skeletal muscle differentiation program [14]–[16]. The four *Mef2* genes exhibit overlapping but distinct expression patterns in embryonic and adult tissues in mice [17] and play a pivotal role in cell differentiation during myogenesis of skeletal, cardiac and smooth muscles. Mef2 proteins are expressed after the myogenic determination factors during mouse embryogenesis and do not seem involved in the initiating events of skeletal myogenesis [17], [18]. Evidence for a role of the Mef2 transcription factors in skeletal myogenesis are essentially coming from cell culture experiments [14], [19], mouse single gene invalidation experiments being not conclusive enough [15], [18], [20], [21], [22].

In *Xenopus*, using a series of gain and loss of function experiments of *Myod*, *Mef2d*, *Paraxis* and *Meox2*, we first showed that the lateral myogenesis appeared to be *Myod*-dependent. *Mef2d* is expressed before *Myod* at the beginning of neurulation in a large domain of the presomitic mesoderm and appears to be an upstream regulator of *Myod* expression in the lateral presomitic cells. Mef2d also drives dermomyotome formation, as evaluated by *Pax3* expression at the tailbud stage. We next identified the dermomyotome progenitors at the beginning of neurulation in the most lateral part of presomitic mesoderm, at the border of lateral myogenic cells. *Mef2d* and *Paraxis (Tcf15)*, a member of the Twist family of transcription factors, are colocalized in this region. Both are necessary for the expression of *Meox2*, a dermomyotome marker in *Xenopus* [23], which acts upstream of *Pax3* expression during dermomyotome formation. Although *Myod* function in lateral myogenesis is conserved between zebrafish and *Xenopus*, the early function of *Xenopus Mef2d* in coupling lateral myogenesis to dermomyotome formation is different from zebrafish and mammals. These results suggest that mechanisms driving somite compartmentalization had dramatically evolved in vertebrates. However, it seems that at least a part of this genetic regulatory network has been used in mammals since *Mef2* acts upstream of *Myod* during mouse skeletal muscle regeneration [24].

## Materials and Methods

### Ethics Statement

This work uses early Xenopus embryos. All experimental procedures used in this study followed the recommendations of the “Comite National de Reflexion Ethique sur l’Experimentation Animale” of the Ministry of Higher Education and Research and were approved by local Animal Care and Use Committees.

### Microinjection of Synthetic mRNA

All pSP64T plasmids used for microinjection were linearized with Xba I. Capped mRNAs were produced *in vitro* from linearized plasmids using the SP6 message machine kit (Ambion). *Xenopus* embryos were injected unilaterally at the two-cell stage at the marginal zone level with 10 to 400 pg of synthetic mRNA and fixed at different stages for whole-mount in situ hybridization or extracted at stage 11 for western blot analysis.

### Morpholino Antisense Oligonucleotide (MO) Injections

Several translation-blocking antisense morpholino oligonucleotides were purchased from Gene Tools (see table 1 and figure S1). In the pseudotetraploïd *Xenopus laevis* species, two genes were cloned for each myogenic factor, *Myod* [25], [26], *Myf5* ([27] and data not shown), *Myogenin* [28], and *Mrf4* [29], [30]. In some cases, mixtures containing two morpholinos directed against each sequence were used: moMyod1 is the 1∶1 mixture of moMyod1a and moMyod1b. moMyf5 is also the 1∶1 mixture of morpholinos a and b. The sequence of moMrf4-1 and -2 is complementary to the two Mrf4 *Xenopus laevis* genes. moMyod1c, moMyod2c, moMyf5c and moMrf4-1c are the mismatch controls of the corresponding oligomorpholinos. To assess the toxicity of MO injections, dose response experiments were performed by unilateral injection of 10 ng to 30 ng of control oligomorpholinos at the two-cell stage. We did not see any phenotypic variation in whole mount in situ hybridization following injection of 30ng control morpholinos per embryo. Injection of moMyod1 or moMyod2 gives rise to the same phenotype. All data presented herein derive from moMyod1. Two genes have also been identified for *Mef2d*, *Paraxis* and *Meox2*. Concerning *Mef2d*, moMef2d1c and moMef2d2c are respectively the mismatch controls of moMef2d1 and moMef2d2. All data presented herein derive from moMef2d1 since the same results were obtained with the two morpholinos. moMef2d1 is the 1∶1 mixture of moMef2d1a and -1b. In the case of *Paraxis* and *Meox2*, all data derived from moParaxis1 and moMeox2-1 respectively. moParaxis2 is the 1∶1 mixture of moParaxis2a and -2b. moParaxis1c and moParaxis2c are respectively the mismatch controls of moParaxis1 and moParaxis2. MoMeox2-1c and moMeox2-2c are respectively the mismatch controls of moParaxis1 and moParaxis2. We also used a morpholino XIMOF8 directed against *Fgf*8 mRNA which has already been characterized [31].

**Table 1:** Sequence of oligomorpholinos.

Oligomorpholino name	sequence
moMyf5a	5′-GATGCTCAGTGGAGTTGAAGCAATC-3′
moMyf5b	5′-CTGACTAGTGCTGTTTAGAAAGATG-3′
moMyf5c	5′-GATcCTCAGTcGAcTTcAAGgAATC-3′
moMyoD1a	5′-GGCAAGAGCTCCATAGAAACAGCCG-3′
moMyoD1b	5′-GGCAACAGCTCCATAGAAACAGCGC-3′
moMyoD1c	5′-GcGAAGAcCTCgATAcAAACAGgCG-3′
moMyoD2a	5′-GATCCACAACTCGGGGGTCCAGGCC-3′
moMyoD2b	5′-CAAAGTCACTACTCCCAGCGAAGGT-3′
moMyoD2c	5′-CAAAcTgACTACTCgCAGgGAAcGT-3′
moMRF4-1	5′-ATAGGTCCATCATTATGCTTTGCCC-3′
moMRF4-1c	5′-ATAcGTgCATgATTATcCTTTcCCC-3′
moMRF4-2	5′-AGCTAAGTGGCGTGCTCAAAACAGC-3′
moMEF2D1a	5′-ATCCTCACTGACCAGCGGAGACCTT-3′
moMEF2D1b	5′-ATCCTCACCGACCAGCGAAGACCTT-3′
moMEF2D1c	5′-ATCgTCAgCGACgAGCcAAGAgCTT-3′
moMEF2D2	5′-CTCCTGCTGGTTCCAGATGTTCTGA-3′
moMEF2D2c	5′-CTgCTGCTGcTTCCAcATcTTgTGA-3′
moparaxis1	5′-AACGGATCATGGTGAAGGCCATGTG-3′
moparaxis1c	5′-AAgcGATCATGcTGAAGcCgATGTG-3′
moparaxis2a	5′-CCATGTGAGCCCTGGGCCAACTCAA-3′
moparaxis2b	5′-CTAGGCTGACTCACTCCTTAATTCC-3′
moparaxis2c	5′-CCATGTcAGCCgTGcGgCAAgTCAA-3′
momeox2-1	5′-CAGGCTTGAGATTCTGGCAGGATTA-3′
momeox2-1c	5′-CAGcgTTGAGATTgTGcCAGcATTA-3′
momeox2-2	5′-GTGTGTGTTCCATGGCATGCAAGTT-3′
momeox2-2C	5′-GTcTGTGTTggATGGCATcCAAcTT-3′
XIMOF8	5′-GGAGGTGATGTAGTTCATGTTGCTC-3′

### Constructs and Directed Mutagenesis

For expression in *Xenopus* embryos, all constructs were subcloned into pSP64T-Flag to generate a C-terminal Flag tagged protein. The pSP64T-Myf5F, -MyodF or -Mrf4F (F for Flag) plasmids have already been described [28]. This is also the case for Mef2dF and Mef2dGRF [32]. ParaxisF, ParaxisGRF, Meox2F and Fgf8b were cloned into pSP64T using “PCR-directed cloning”, a method developed in our laboratory [32] and by [33]. All these plasmids contain the 5′- and 3′-UTR of globin gene, the coding sequence of the corresponding gene and the coding sequence of Flag epitope (except for Fgf8b). To test the activity of morpholinos, pSP64T plasmids containing the 5′-UTR of globin were replaced by the 5′-UTR of Myf5a or b, Myoda or b, Mrf4, Mef2da or b, Paraxis and Meox2 using PCR-directed cloning. These plasmids were named pSP64T-5utrMyf5F, -5utrMyodF, -5utrMrf4F, –5utrMef2dF, -5utrMeox2F and -5utrParaxis. pSP64T-5utrMyf5, Myod and Mef2d are the 1∶1 mixture of a and b forms. In order to construct rescue plasmids coding for a synthetic *Paraxis* mRNA which must be not recognized by moParaxis1, part of the sequence complementary to the morpholino was mutated using the quick change mutagenesis method (Stratagene, Agilent technologies). These constructs, named pSP64T*Paraxis*F’, present 10 mismatches in the nucleotide sequence recognized by the corresponding morpholinos. For immunoprecipitation experiments, *Mef2d* was subcloned into pcDNA3.1/V5/HIS (Invitrogen) and *Paraxis* into pBIND (Promega) vectors using PCR-directed cloning. For luciferase assay, *Xenopus tropicalis Meox2* promoter (-907 to +80) [34] was cloned into pGl4 vector (Promega) by PCR-directed cloning and named Pgl4p*Meox2*-luc. Moreover, pBIND-Gal4Paraxis was deleted of luciferase and Gal4 domains using the quick change mutagenesis method and named pbind’-Paraxis.

### Western Blot

Western blotting experiments with anti-Flag antibodies have already been described [35].

### Whole-mount in situ Hybridization, Immunohistochemistry and Embryo Sections

cDNA for whole-mount in situ hybridization probes were prepared by RT-PCR (primers presented in table S1). Probes were carried out as previously described [29]. Double whole-mount in situ hybridizations were performed with fluorescein-labeled and digoxigenin-labeled RNA probes, and two successive color reactions (BCIP, Sigma-Aldrich, Saint-louis, MO and BM-purple, Roche applied science, Mannheim, Germany) separated by heat inactivation (65°C, 30 min) of alkaline phosphatase. For transversal sections, embryos were fixed 2 hours in MEMFA, embedded in 4% agarose, and sectioned at a thickness of 40 µm with a vibratome. For whole-mount immunohistochemistry, the concentrated muscle-specific 12/101 monoclonal antibody from Developmental studies hybridoma bank (University of Iowa), was used (1∶2000). The primary antibody was either detected with an alkaline phosphatase-conjugated anti-mouse antibody (Vector Laboratories) diluted at 1∶2000 followed by reaction with BM-purple or Magenta phosphate (Sigma-Aldrich) or with Alexa fluor donkey 488 anti-mouse secondary antibody (Life technologies). In cases where both in situ hybridization and 12/101 staining were carried out, embryos were first stained using in situ hybridization, immediately followed by immunohistochemistry.

### Lineage Tracing Experiments using Fluorescent Tracers

Stage 13 embryos were injected using pulled-glass capillaries either with WGA-rhodamine (dissolved at 200 µg/ml, Vector laboratories) to be analysed at stage 18 or with both WGA-rhodamine and -fluorescein (Vector laboratories) to be analysed at stage 23. Injections with WGA-rhodamine and -fluorescein were made at the most lateral level and in the lateral level of the trunk presomitic mesoderm respectively. All embryos were photographed at the beginning of the experiment, then incubated for 5 to 10 hours at 22°C until stage 18 or 23 and fixed in 4% formaldehyde. The fate of stained cells was directly analyzed on 50 µm transversal vibratome sections at the trunk level (stage 23) or after immunofluorescent experiment with the 12/101 antibody and Alexa fluor donkey 488 anti-mouse secondary antibody (stage 18). The experiment was repeated six times to verify that similar experimental results were obtained.

### Microdissection Experiments

Dechorionated embryos were oriented and immobilized in 0.4XMMR 1% agarose gel slits. Using sharpened needles, anteroposterior incision in the ectoderm was realized at stage 14 on the dorsal side of the embryo at the mediolateral or lateral level. Ectoderm and mesoderm were separated from each other on the lateral incision side and the superficial part of the mesoderm was removed. The sham-operated embryos were treated without removing the mesoderm. Embryos were fixed immediately or following development until stage 19 or 30–32. Embryos were analysed by whole mount in situ hybridization.

### Inhibition of Fgf Receptor Signalling at the End of Gastrulation

Normal embryos or embryos unilaterally injected at the two-cell stage with Mef2dF mRNA were cultured until stage 12. 20 nanoliters of DMSO solution or a 5 mM DMSO stock of the Fgf receptor kinase inhibitor SU5402 (Calbiochem) were injected into the archenteron. Embryos were cultured until stage 15/16 and were processed for in situ hybridization.

### Cycloheximid and Dexamethasone Treatment

To induce glucocorticoid fusion variants, embryos were treated with 10 µM dexamethasone (DXM; Sigma) at stage 12.5–13 for 2 hours or until the tailbud stage. To block protein synthesis, embryos were incubated with 20 µg/ml cycloheximid (CHX; Sigma) in 0.4XMMR for 45 min [36], [37] and then treated for hormone induction of glucocorticoid fusion variants with 10 µM DXM for 2 hours.

### Transfections, Coimmunoprecipitation and Luciferase Assays

Cell culture of COS7 cells was described previously [38]. Transfections or cotransfections of pGl4p*meox2*-luc, pbind’-Paraxis and pcDNA3-Mef2dV5/HIS were performed in 24-well plates using FuGENE 6 (Roche Applied Science) and luciferase activity was measured 48h after transfections using the dual luciferase system (Promega). Transfection assays were repeated 4 times. For co-immunoprecipitation assays, transfections or cotransfections of pBIND, pBIND-Paraxis and pcDNA3-Mef2dV5/HIS were realized in 6-well plates and protein were extracted 48h after transfections. V5/His tagged Mef2d constructs were immunoprecipitated using Ni-NTA beads (Invitrogen), followed by western blotting procedures. Antibodies used included mouse monoclonal anti-V5 (Invitrogen) and mouse monoclonal anti-Gal4 (Santa Cruz).

## Results

### Gene Knock Down Experiments Reveal that Myod is Critical for *Xenopus* Lateral Myogenesis

**Figure 1:**
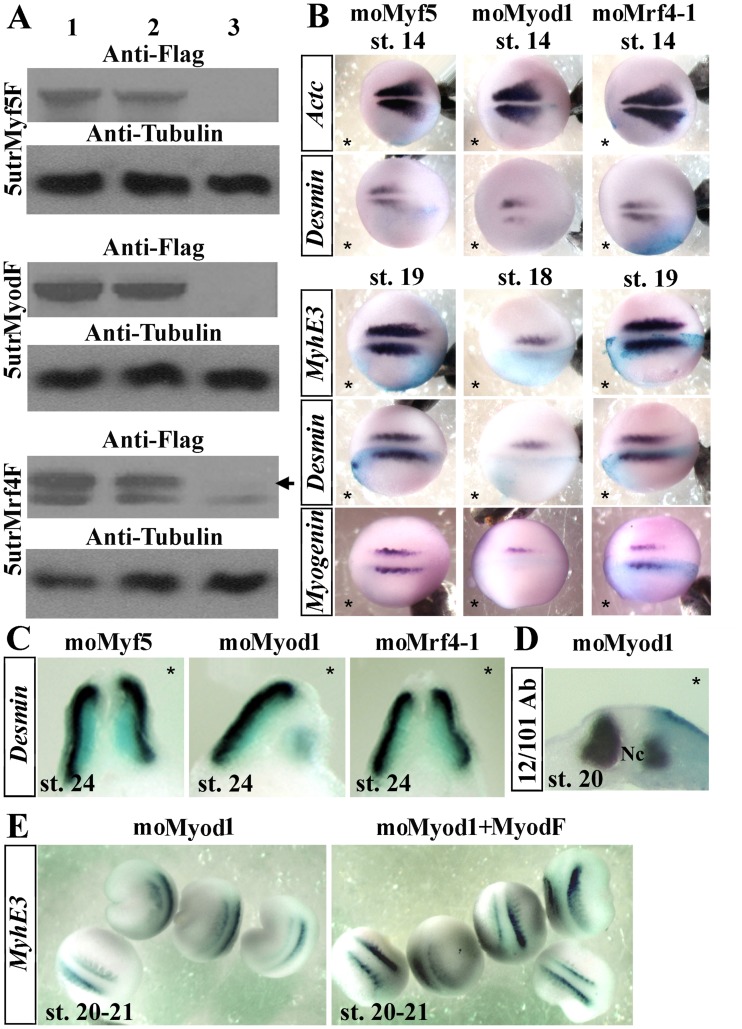
Myod is required for lateral myogenesis. (A) Western blot with anti-flag and anti-tubulin antibodies of gastrula embryos injected bilaterally at the two-cell stage with 300 pg of 5utrMyf5F, 5utrMyodF or 70 pg of 5utrMrf4F synthetic mRNAs alone or with oligomorpholinos. Arrows point out the specific signal. Lane 1: synthetic mRNA alone, 2: synthetic mRNA+moControl, 3: synthetic mRNA+specific mo (moMyf5, moMyod1 or moMrf4-1 with 5utrMyf5F, 5utrMyodF or 5utrMrf4F respectively). (B) Whole-mount in situ hybridization of embryos unilaterally injected with 20 ng of moMyf5, moMyod1 or moMrf4-1 and fixed at stages 14 or 18/19. β-galactosidase mRNA (blue) was co-injected to identify the injected side, indicated by an asterisk (*). Dorsal views. The anterior side of the embryos is on the left; st., stage. (C) Transverse sections of the morphants at stage 26. (D) Transverse section of the *Myod* morphant submitted to whole-mount immunohistochemistry with the 12/101 antibody. (E) Rescue experiments: Embryos were injected unilaterally with 20 ng of mo*Myod*1 alone or co-injected with synthetic mRNA coding for MyodF (150 pg) and probed with *MyhE3*. Nc, notochord. Probes are in a framed box and indicated for each panel. For complete statistical data, see supporting information, figure S2.

In *Xenopus,* lateral myogenesis is characterized by a myogenic program expressing *Myod* at a strong level, *Myogenin* at moderate levels and *Mrf4*. In contrast, the medial cell population expresses a different combination of MRF: *Myf5*, *Myod* and *Mrf4* [6]. We first studied the role of the three determination MRFs using gene knockdown experiments to identify essential factors involved in lateral myogenesis. Translation was efficiently inhibited by injections of the blocking MO (moMyod1, moMyf5, moMrf4-1) and the corresponding mRNA coding for the flag-tagged MRF (Fig. 1A, lane 3). Then, embryos were unilaterally injected with 20 ng of MO at the two-cell stage and differentiation of the medial myogenesis was analyzed at the beginning of the neurulation (stage 14) (Fig. 1B). The three MRF morphants showed little variation in *Actc* (Cardiac α-actin) expression, whereas *Myod* morphants displayed a slight decrease in *Desmin* expression in 50% of the analyzed embryos (n = 62). At later stages, only *Myod* morphants displayed a sharp decrease in *Desmin* (73.3%, n = 70), *MyhE3* (70%, n = 50) and *Myogenin* (71.4%, n = 56) expression (Fig. 1B). *Desmin*, *MyhE3*, and *Myogenin* expression at this stage correspond to the differentiation of lateral myogenesis [6]. Transverse sections of somites at stage 26 indicated that the myotome development is specifically affected in *Myod* morphants (Fig. 1C). Staining with the muscle specific 12/101 antibody on transverse sections at stage 22 confirmed that only the lateral myogenesis is affected in *Myod* morphants (70%, n = 10) since the first differentiated myogenic cells of medial myogenesis, located near the notochord [6] remains strongly stained (Fig. 1D). Mismatch control MOs had no effect (see supporting information). Furthermore, the specificity of *Myod* oligomorpholino was corroborated by a partial rescue of *MyhE3* expression (63.3%, n = 60) when co-injected with *Myod* mRNA (Fig. 1E) and was confirmed by another set of translation blocking MO against *Myod* (moMyod2) and *Mrf4* (moMrf4-2) (see supporting information). Previous knock down experiments carried out with another *Myf5* translation blocking MO also showed no drastic effect on myogenesis [39]. Altogether these results showed that *Myod* is required for the lateral myogenesis in *Xenopus*, as supported by previous works in zebrafish [5].

### Mef2d is Required for Lateral Myogenesis by Activating *Myod* Expression

**Figure 2:**
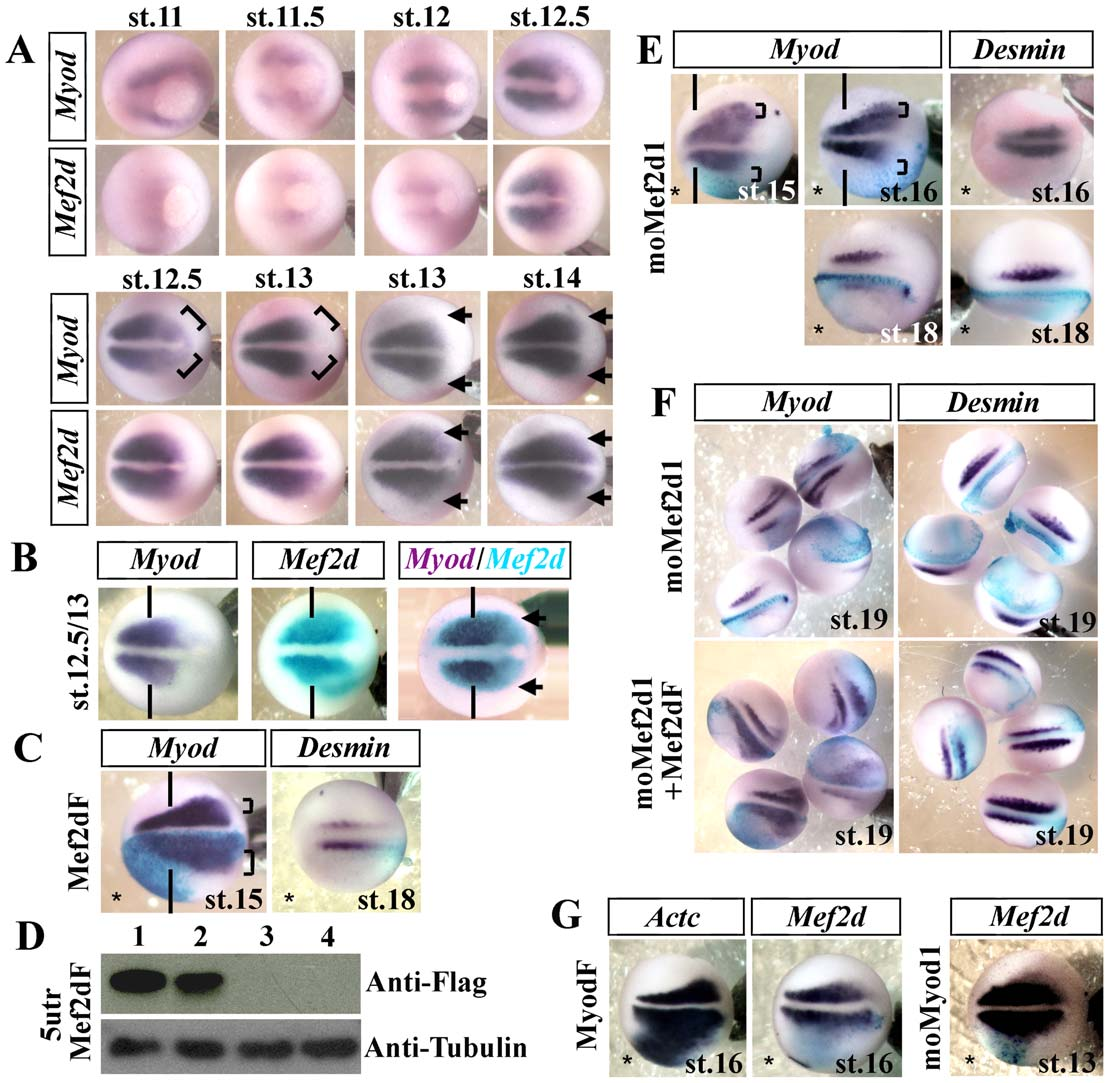
*Mef2d* drives lateral *Myod* expression. (A) In situ hybridization of *Myod* and *Mef2d* at gastrula and neurula stages realized from time-course experiment with embryos of the same fecondation pool. Brackets show initial posterior *Myod* expression. (B) Single and double *in situ* hybridization with *Mef2d* (blue) and *Myod* (purple) show that *Mef2d* precedes *Myod* expression in the posterolateral domain of presomitic mesoderm (arrows) at stage 12.5/13. (C) Embryos injected unilaterally with 400 pg of Mef2dF mRNA. (D) Western blot with anti-flag and anti-tubulin antibodies of late gastrula embryos injected bilaterally either with 300 pg of 5utrMef2dF synthetic mRNA alone (lane1) or with oligomorpholinos: moControl (lane 2), moMef2d1 (lane3), or moMef2d2 (lane 4). (E) Embryos injected unilaterally with 20 ng of moMef2d1 and submitted to in situ hybridization to detect either *Myod* or *Desmin* mRNA. (F) Co-injection of Mef2dF mRNA (200 pg) with moMef2d1 was able to rescue the phenotype of moMef2d1 embryos. (G) Unilateral injection of MyodF mRNA (300 pg) or moMyod1 and detection of *Actc* and *Mef2d* expression at stage 16. β-galactosidase mRNA (blue) was co-injected to identify the injected side, indicated by an asterisk (*). Vertical lines define the limit between anterior and trunk regions. Probes are in a framed box and indicated for each panel. Dorsal views. the anterior side of the embryos is on the left; st., stage. For complete statistical data, see supporting information, figure S2.

Next, we were interested in the members of the Mef2 family of transcription factors which cooperate with Myod during myogenesis. *Mef2a* and *Mef2d* are expressed during the first wave of *Xenopus* myogenesis [32]. *Mef2a* expression has the profile of a differentiation gene as *Desmin* or *Mrf4* [6]; It begins to be expressed at stage 13 in paraxial mesoderm (data not shown). In contrast, *Mef2d* expression [32] is similar to *Myod* one [6], [40] and it was early detected by whole-mount in situ hybridization in presomitic mesoderm from gastrula at stage 11 [32] suggesting that *Mef2d* could play an early role. Therefore, we compared *Mef2d* expression with *Myod* one. During gastrulation, *Myod* expression is concentrated around the blastopore and in the medial region of paraxial mesoderm (Fig. 2A, st. 11 to 12). At the gastrulation-neurulation transition time, the residual expression of *Myod* in the posterior region disappears (Fig. 2A, st. 12.5–13, brackets) before its new extension in the lateroposterior region (Fig. 2A, st. 13–14). These results were in accordance with the previously described *Myod* mRNA expression pattern at stage 12.5/13 [41] and 13/14 [42]. At mid-gastrulation *Mef2d* expression is faint and localized inside the anterior *Myod* expression domain (Fig. 2A, st. 11 to 12). At the gastrulation-neurulation transition time, *Mef2d* expression begins to be strong and expends lateroposteriorly before *Myod*. (Fig. 2A, st. 13–14, arrows and Fig. 2B, arrows). *Myod* has been identified as a Mef2 target gene in *Xenopus* [43], [44], therefore we investigated *Mef2d* function in the lateral myogenesis using gain and loss of function experiments. Injection of *Mef2d* mRNA (Mef2dF) induced a lateral enlargement of the *Myod* expression domain (68.4%, n = 38) at stage 15 (Fig. 2C, brackets) and enhanced *Desmin* expression (70.3%, n = 37) at stage 18 (Fig. 2C). For knockdown experiments, we used the same procedure as for MRF oligomorpholinos to test the specificity and the efficiency of translation inhibition (Fig. 2D). The *Mef2d* morphants displayed no *Myod* mRNA expression (67.7%, n = 62) in the lateral presomitic mesoderm at stage 16 (Fig. 2E, brackets). Differentiation of the lateral myogenic wave was also strongly affected by the moMef2d1 morpholino injection since *Desmin* (Fig. 2E) and *MyhE3* (data not shown) were not expressed at stage 18, (70.2%, n = 57) although *Desmin* expression of the medial myogenesis remained unaffected (72.1%, n = 49) at stage 16 (Fig. 2E). Rescue experiments totally restored *Myod* expression (75%, n = 60) in the lateral presomitic mesoderm and partially (70.6%, n = 68) *Desmin* (Fig. 2F) and *MyhE3* mRNA (data not shown) expression in somites. Since a feed-forward loop initiated by Myod appears to be involved in the regulation of *Mef2* expression [45], [46], we tested whether Myod may induce *Mef2d* expression. *Myod* mRNA (MyodF) injection resulted in a strong ectopic expression of *Actc* (78.%, n = 38), without significantly altering *Mef2d* (100%, n = 40) expression (Fig. 2G). Likewise, moMyod1 had no effect on *Mef2d* expression (70%, n = 44) in presomitic mesoderm (Fig. 2G). In conclusion, Mef2d controls *Myod* expression during *Xenopus* lateral myogenesis.

### Mef2d is Necessary for the Activating Effect of Fgf on Lateral *Myod* Expression at the Early Neurula Stage

**Figure 3:**
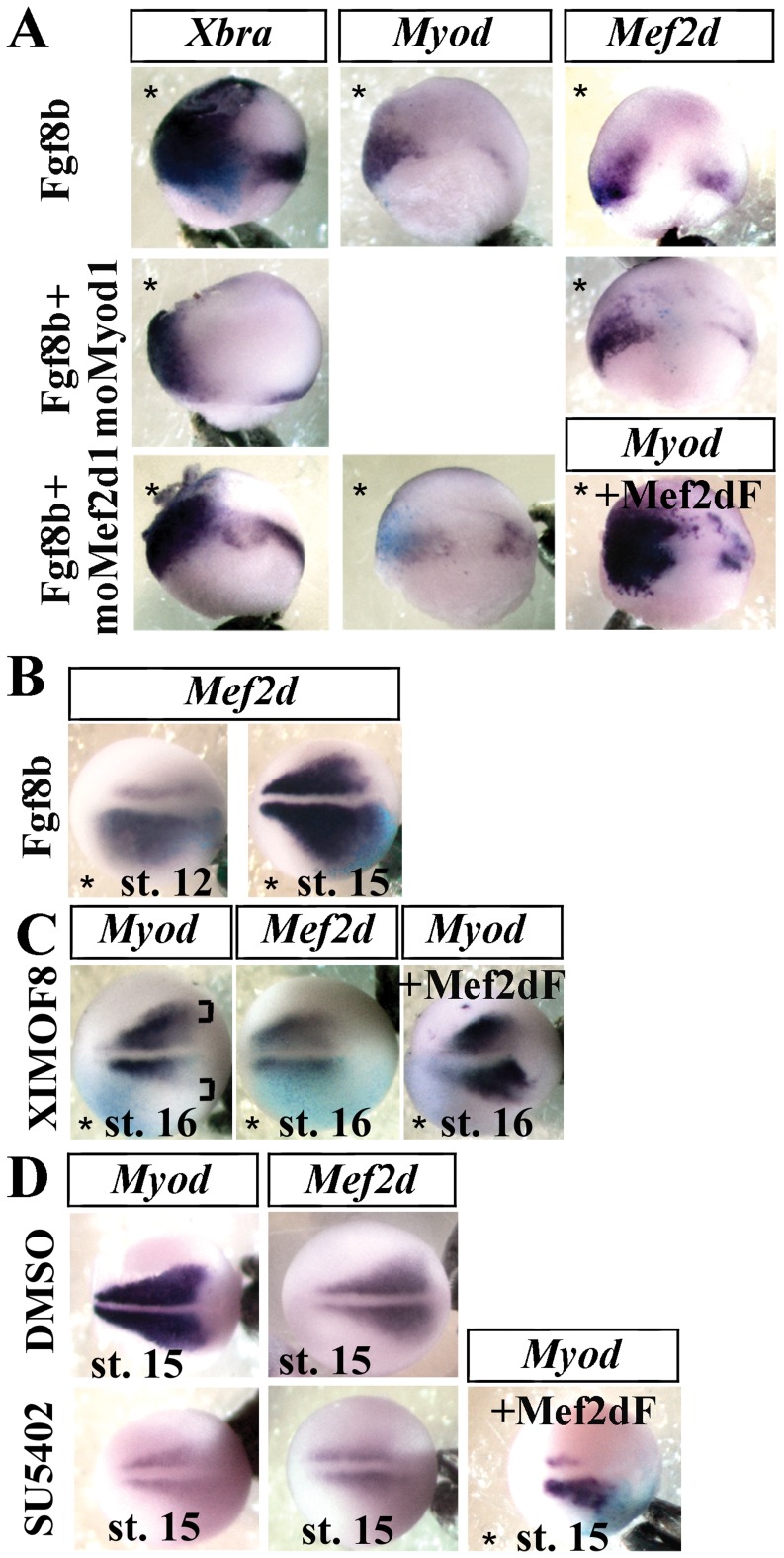
Mef2d is necessary for the activating effect of Fgf on *Myod* expression at the neurula stage. (A) Embryos were injected unilaterally either with Fgf8b synthetic mRNA alone (5 pg), or with moMyod1 or with moMef2d1, fixed at the gastrula stage and submitted to in situ hybridization for *Xbra*, *Myod* or *Mef2d*. Co-injection of Mef2dF mRNA with moMef2d1 was able to rescue the phenotype of moMef2d1 embryos (+Mef2dF). (B) Unilateral injection of Fgf8b mRNA induced the lateral expansion of *Mef2d* expression domain at stage 16. (C) Embryos were injected unilaterally with 20 ng of XIMOF8 and fixed at stage 16. Co-injection with Mef2dF mRNA was able to rescue the phenotype of XIMOF8 embryos (+Mef2dF). (D) Embryos were injected at stage 11.5/12 with DMSO or 5 mM SU5402, an inhibitor of Fgf signaling, and fixed at stage 16. A first unilateral injection of Mef2dF mRNA at the two-cell stage was able to rescue the phenotype of treated embryos (+Mef2dF). Injected side (*) at the bottom except in (A), on the left. Bracket indicates the position of lateral myogenic cells. Probes are in a framed box and indicated for each panel. For complete statistical data, see supporting information, figure S2.

In *Xenopus*, Fgf is a major player in the mesoderm maintenance [47], [48]. In addition, Fgf induces *Myod* expression in an animal cap assay [49] and, like Mef2d, leads to a lateral enlargement of *Myod* expression domain [31], [50]. In particular, *Fgf*8 is strongly expressed in the posterior mesoderm during neurulation [31], [51] and is involved in lateral fast myogenesis in zebrafish [4]. Here, unilateral injections of *Fgf*8 at the two-cell stage not only induced *Xbra (T)* (85.5%, n = 35) and *Myod* overexpression (73.7%, n = 38), as already reported [31], but also that of *Mef2d* (69.4%, n = 36) (Fig. 3A and B). In the presence of moMef2d1, Fgf8 activation of *Myod* transcription was abolished (60%, n = 40); it was rescued (69.2%, n = 39) by co-injection with Mef2dF (Fig. 3A). Fgf8 induction of *Mef2d* was not affected (75%, n = 48) by moMyod1 (Fig. 3A). Conversely, the *Fgf*8 morphants, injected unilaterally with the characterized oligomorpholino XIMOF8 [31], showed not only a loss of *Myod* (69%, n = 42) expression (Fig. 3C, brackets), but also a loss of *Mef2d* expression (63.8%, n = 58) in the lateral presomitic mesoderm (Fig. 3C). The *Myod* expression domain was rescued by co-injection (68.2%, n = 44) with *Mef2d*F (Fig. 3C). Moreover, the injection of a Fgf receptor inhibitor (SU5402) in the archenteron cavity at stage 11.5/12 results in the loss of lateral expression of *Myod* (73.6%, n = 53) and *Mef2d* (63.8%, n = 52) at stage 16 (Fig. 3D). A previous unilateral injection of Mef2dF at the two-cell stage partly rescued *Myod* (68.7%, n = 48) expression (Fig. 3D). Altogether, these data demonstrate that *Mef2d* is necessary for the activating effect of Fgf on the lateral *Myod* expression domain at the neurula stage.

### Mef2d Drives the Formation of the Dermomyotome

**Figure 4:**
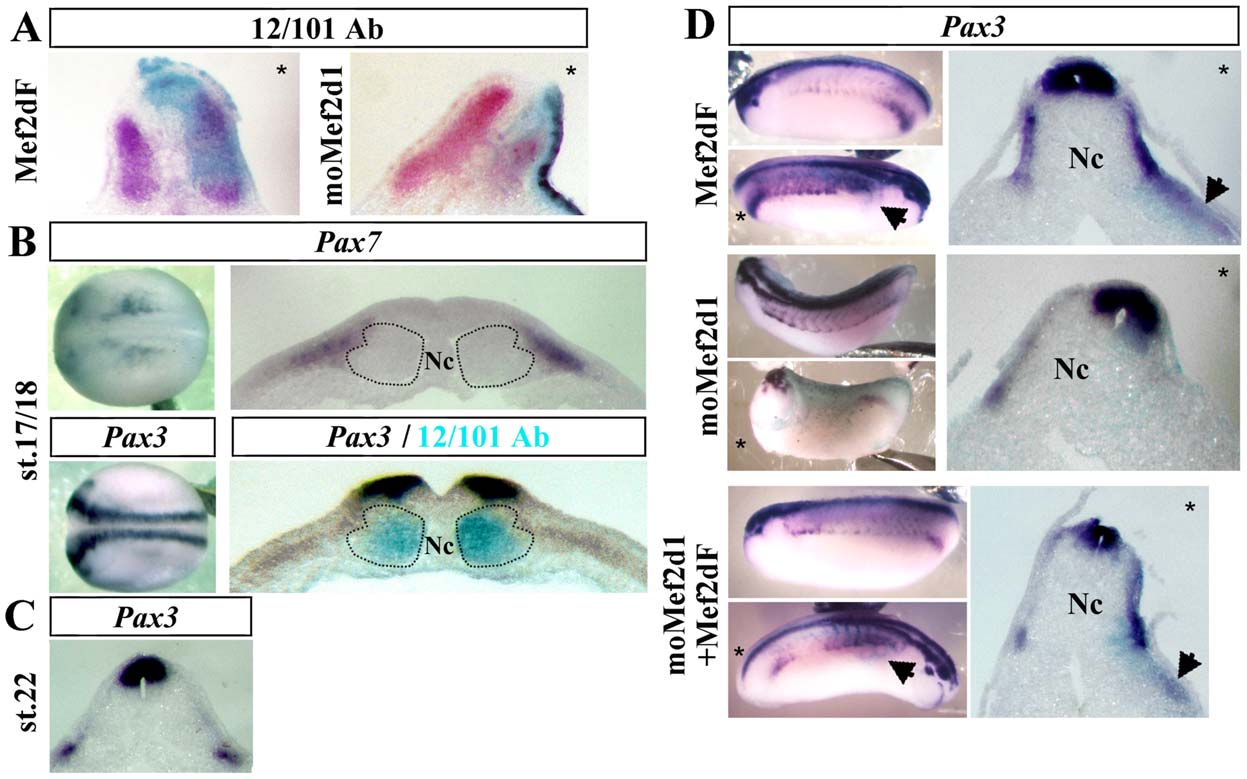
Mef2d is required for dermomyotome formation. (A) Embryos injected unilaterally with 400 pg of Mef2dF mRNA or 20 ng of moMef2d1 were fixed at stage 26 and submitted to staining with the specific muscle 12/101 antibody (red). (B) Expression of *Pax7* and *Pax3* mRNA at stage 17/18 (dorsal view or transverse sections at the trunk level). Co-staining of *Pax3* mRNA (purple) and differentiated muscle cells detected by 12/101 antibody (blue). Dotted lines indicate the position of the medial and lateral population of myogenic cells [5]. (C) Expression of *Pax3* mRNA on a transverse section at stage 22. (D) *Pax3* mRNA expression on lateral view (left) or on transverse section (right) at the tailbud stage after unilateral injection of Mef2dF or moMef2d1. Rescue experiments restored the phenotype of moMef2d1. Arrows indicate the sites of lateral ectopic expression of *Pax3*. (*) Injected side. Probes are in a framed box and indicated for each panel. Nc, notochord. For complete statistical data, see supporting information, figure S2.

The size of the muscle compartment stained with the 12/101 antibody at stage 25–26 was enlarged in the dorsal somitic region of embryos injected with Mef2dF (66.6%, n = 9) and decreased in *Mef2d* morphants (75%, n = 8), particularly in the hypaxial region, confirming that Mef2d promotes myogenesis (Fig. 4A). As the most lateral cells of the paraxial mesoderm express *Mef2d* at the beginning of neurulation (Fig. 2A) and give rise later to the most dorsal somitic structures [6], we hypothesized that Mef2d could also be involved in dermomyotome formation, which was analyzed by *Pax3* expression at tailbud stages 28–32. *Pax3* [8] like *Pax7* [52] begins to be expressed in the most dorsolateral region of the somites from stage 17/18 (Fig. 4B). At stage 22/23, *Pax3* expression is restricted to dermomyotome (Fig. 4C), especially in the hypaxial region. A Mef2dF injection induced an increase of *Pax3* expression (66%, n = 94) in the dorsal somitic region and, in one third of the embryos, a faint ectopic *Pax3* expression in the lateral mesoderm (Fig. 4D, arrows). Conversely, *Mef2d* morphants exhibited a decrease of *Pax3* expression (71.4%, n = 49) that was restored in rescue experiments (67.3%, n = 58). In addition, one third of the rescued embryos showed an increase of *Pax3* expression in somites and an ectopic expression in the lateral mesoderm (Fig 4D, arrows). Altogether, these results demonstrate that Mef2d promotes *Pax3* expression during dermomyotome formation.

### *Mef2d* Cooperates with *Paraxis* to Promote Dermomyotome Formation

**Figure 5:**
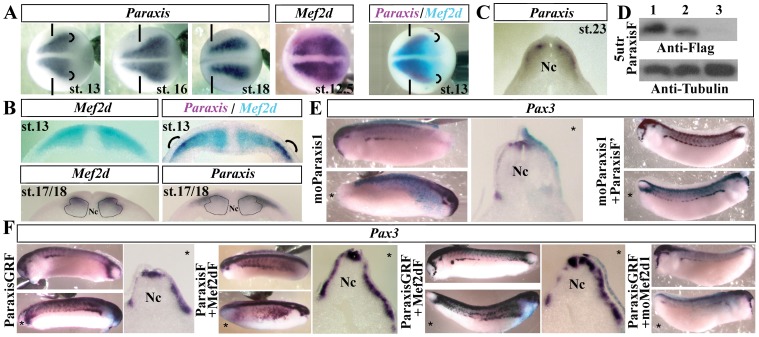
Paraxis and Mef2d have a cooperative effect on dermomyotome formation. (A) Expression of *Paraxis* mRNA during neurulation. Costaining of *Mef2d* (blue) and *Paraxis* (purple) mRNA at stage 13 in comparison with *Mef2d* expression alone. Rounded brackets indicate the region of colocalization of *Paraxis* and *Mef2d*. Dorsal views. The anterior side of the embryos is on the left; st., stage. Vertical lines define the limit between anterior and trunk region. (B) Transverse sections of the costained embryos compared to *Mef2d* staining at stage 13 (upper panels). Expression of *Paraxis* and *Mef2d* at stage 17/18 (lower panels). Dotted lines indicate the position of the medial and lateral population of myogenic cells. (C) Expression of *Paraxis* mRNA on a transverse section at stage 23. (D) Western blot with anti-flag and anti-tubulin antibodies of late gastrula embryos injected bilaterally either with 300 pg of 5utrParaxis synthetic mRNA alone (lane1) or with oligomorpholinos: moControl (lane 2) or moParaxis1 (lane3). (E) Embryos injected unilaterally with 20 ng of moParaxis1 were submitted to *in situ* hybridization with *Pax3* antisense probe at the tailbud stage (lateral view or transverse section). A co-injection of ParaxisF’ mRNA (150 pg) with moParaxis1 was able to rescue the phenotype of moParaxis1 injected embryos (lateral view). (F) Unilateral injection of ParaxisGRF (150 pg) induced an increase of *Pax3* mRNA expression at the tailbud stage. Pax3 expression after co-injection of ParaxisF+Mef2dF or ParaxisGRF+Mef2dF. ParaxisGRF injection with moMef2d1 had no effect on *Pax3* expression. (*) Injected side. Probes are in a framed box and indicated for each panel. Nc, notochord. For complete statistical data, see supporting information, figure S2.

As injections of Mef2dF only slightly affect *Pax3* expression, we looked for a partner which could cooperate with Mef2d to induce *Pax3* expression. Paraxis, a bHLH transcription factor of the Twist family, is a good candidate for several reasons. First, *Paraxis* is necessary for hypaxial myogenesis and dermomyotome formation in mouse [53]. Second, we have shown previously by gain of function experiments that another member of the Twist family, *Scx* (*Scleraxis*), cooperates with another member of the Mef2 family, *Mef2c,* to induce the expression of the tendon structural genes *Tgfbi* (*beta-IgH3*) and *Tnc* (*tenascin-C*) [31]. In *Xenopus*, *Paraxis* is expressed in somites [54], particularly in the most lateral part of paraxial mesoderm (Fig. 5A and B, st. 13, rounded brackets), co-localizing with *Mef2d* at stage 13 (Fig. 5A and B, st. 13). At stage 17/18, *Paraxis* is expressed in the most dorsolateral part of the somites, (Fig. 5 B, st. 17/18), a region where *Pax3* and *Pax7* are also expressed (Fig. 4B). This observation is confirmed at stage 23 (Fig. 5C). To identify the role of *Paraxis,* loss of function experiments were realized. The efficiency of translation inhibition was demonstrated by successive injections of a blocking MO (moparaxis1) and the mRNA coding for flag-tagged Paraxis (Fig. 5D). An injection of moParaxis1 induces a decrease of *Pax3* expression (71.7%, n = 68) at the tailbud stage, and this phenotype was rescued by injection of ParaxisF’ (79%, n = 61) (Fig. 5E). Overexpression experiments resulted in an increase of *Pax3* expression. While only 20% of the embryos displayed this phenotype with ParaxisF (data not shown), an inducible ParaxisGRF injection resulted in *Pax3* overexpression in 75% of the embryos (n = 62) (Fig. 5F). As both transcription factors, Mef2d and Paraxis, modulate *Pax3* expression, we tested a putative cooperative effect and found that *Pax3* expression was strongly enhanced in dermomyotome and extended to the ventral region in 20% of the embryos co-injected with Mef2dF and ParaxisF and in 70% of the embryos co-injected with Mef2dF and ParaxisGRF (n = 40) (Fig. 5F). As expected, injections of moMef2d1 abolished Pax3 overexpression (11.3%, n = 62) induced by a ParaxisGRF injection (Fig. 5F).

### Mef2d and Paraxis Target *Meox2* Gene in the Dermomyotome Progenitors at the Beginning of Neurulation

**Figure 6:**
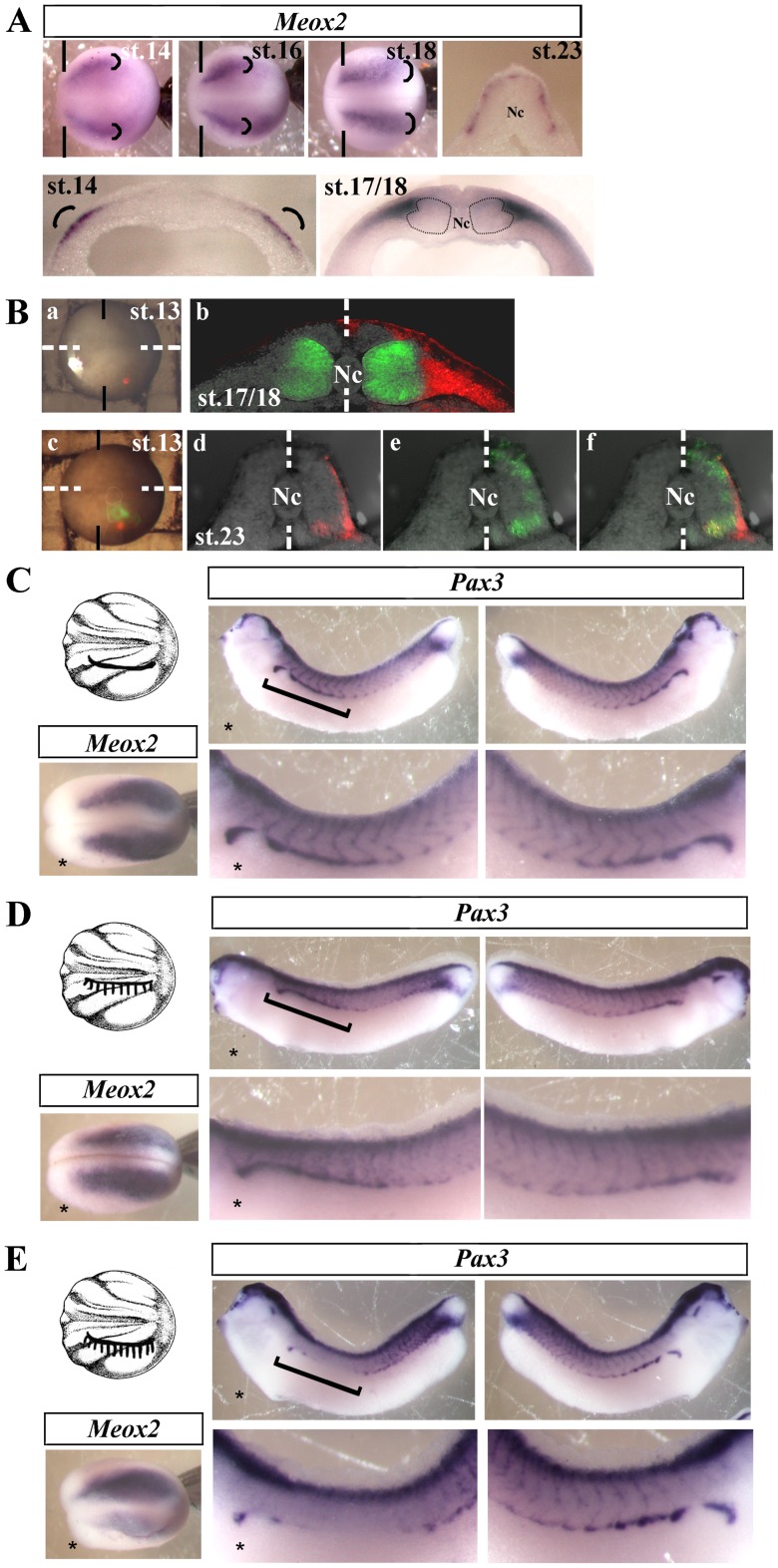
Lateral presomitic cells expressing *Meox2* are the dermomytome progenitors. (A) Expression of *Meox2* mRNA in the most lateral region (rounded brackets) of presomitic mesoderm at stage 14 (dorsal view and transverse section), 16 (dorsal view), 17/18 (dorsal view and transverse section) and 23 (transverse section). Dotted lines indicate the position of the medial and lateral population of myogenic cells. Vertical lines define the limit between anterior and trunk region. Nc, notochord. (B) Lineage tracing experiments: embryos were injected with WGA-rhodamine (red) in the most lateral presomitic mesoderm (a) or co-injected with WGA-fluorescein (green) in the lateral presomitic mesoderm (c) at stage 13. Transverse sections of the embryo at the trunk level and at stages 18 or 23; embryo injected with WGA-rhodamine and submitted to indirect immunofluorescence with 12/101 antibody followed by secondary Alexa fluor 488 anti-mouse antibody (green) (b) or injected with the both tracers (d, e and f). WGA-rhodamine fluorescence (d), WGA-fluorescein fluorescence (e), merge (f). Dotted lines indicate the bilateral symmetry plan. In (a) and (c): Vertical lines define the limit between anterior and trunk region. (a) and (c) dorsal views, anterior side on the left. (C, D and E). Ablation experiments of presomitic mesoderm at stage 14. After microdissection experiments, embryos were fixed at stage 19 or at the tailbud stage to analyze meox2 and pax3 expression respectively. (C) sham-operated embryos, ectoderm was incised at the lateral level (line), ectoderm and mesoderm were separated from each other on the lateral side, but mesoderm was not removed. (D) Same operation on the mediolateral level but superficial mesoderm was removed (hatched zone) (E) Same operation on the lateral level but superficial mesoderm was removed (hatched zone). Brackets show the axis level corresponding to incision. For complete statistical data, see supporting information, figure S2.

**Figure 7:**
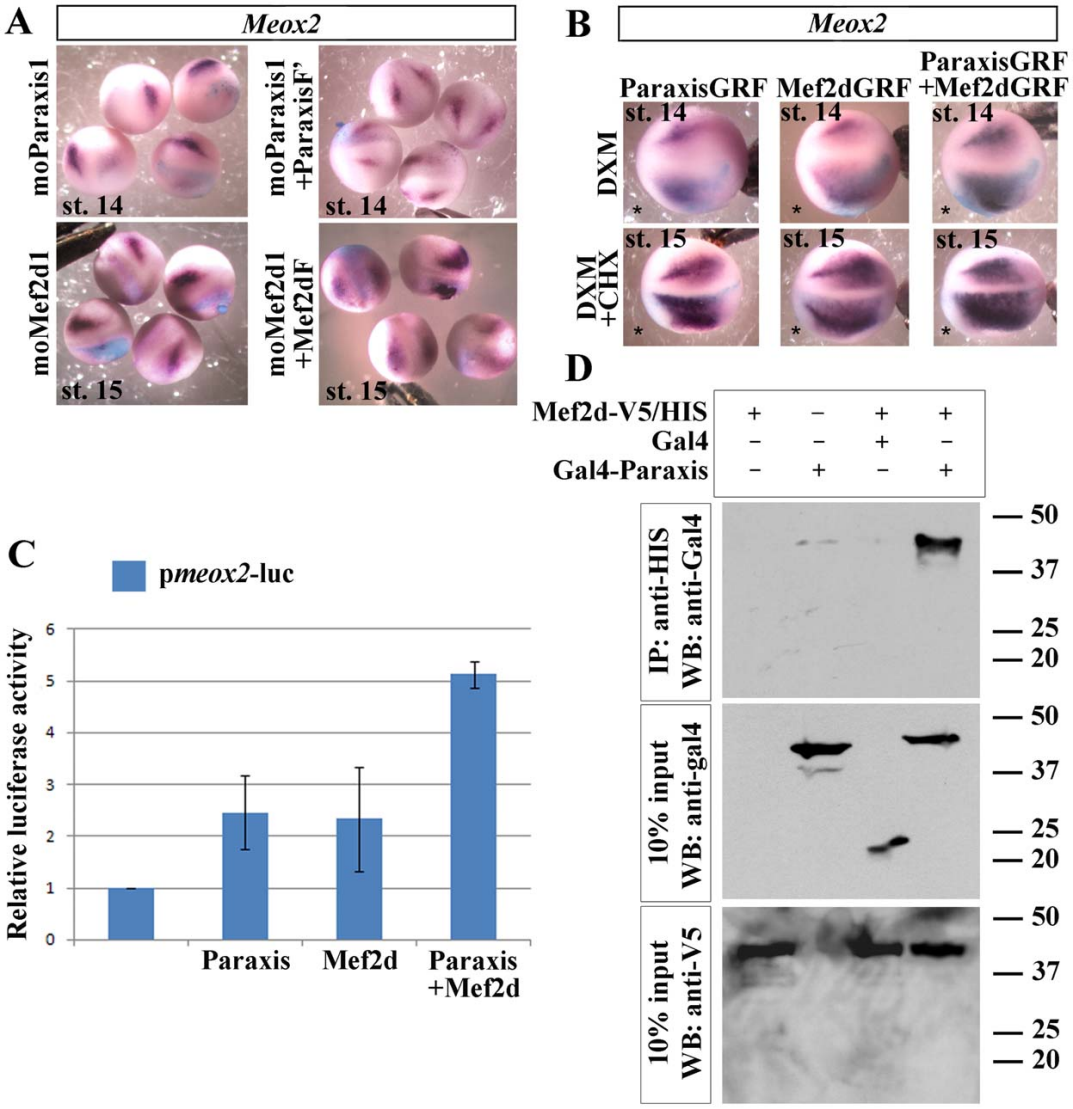
Paraxis and Mef2d targets *Meox2* gene in the most lateral part of the presomitic mesoderm. (A) Embryos injected unilaterally with 20 ng of moParaxis1 or moMef2d1 were submitted to *in situ* hybridization with *Meox2* antisense probe at the neurula stage. Injection of either ParaxisF’ mRNA with moParaxis1 or Mef2dF with moMef2d1 was able to rescue the phenotype. (B) Unilateral injection of either ParaxisGRF or Mef2dGRF induced an increase of *Meox2* mRNA expression at the neurula stage after induction by dexamethasone (DXM) at stage 12.5. A cooperative effect was observed after co-injection of ParaxisGRF and Mef2dF. A treatment by cycloheximid (CHX) followed by induction by dexamethasone (DXM) at stage 12.5 indicated that *Meox2* is a direct target gene of Paraxis and Mef2d. (*) Injected side. Probes are in a framed box and indicated for each panel. (C) COS7 cells were transfected with p*meox2*-luc alone, or co-transfected with either Paraxis, Mef2d-V5/His or both and luciferase activity was determined 48 h after transfection. * P<0.01 (D) Protein extracts from COS7 cells transfected with Mef2d-V5/His alone or with either empty vector (Gal4) or Gal4-Paraxis construct were immunoprecipitated (IP) with Ni-NTA beads and subjected to Western blot (WB) using an anti-Gal4 antibody (upper panel). Input control experiments with anti-V5 (lower panel) or anti-Gal4 (mid panel) antibodies. For complete statistical data, see supporting information, figure S2.

At the beginning of neurulation, *Mef2d* co-localizes with *Paraxis* in the most lateral part of paraxial mesoderm, a region probably corresponding to the precursors of dermomyotome (Fig. 5A and B, st. 13). From stage 17/18, *Mef2d* expression profile (Fig. 5B, st. 17/18) is no longer expressed in the most dorsolateral part of the somites where *Pax3* begins to be expressed (Fig. 4B). Therefore, the effect of Mef2d on *Pax3* expression should be indirect. Thus, we looked for a direct target gene of Mef2d and Paraxis in the progenitors of dermomyotome. Among genes involved in somite formation, *Meox2* is expressed in these *Mef2d*/*Paraxis* expressing cells at stage 14 (Fig. 6A, st. 14, rounded brackets). Later, at stage 17/18, *Meox2* is present in the most lateral region of the somites (Fig. 6A, st. 17/18) where *Pax3* and *Pax7* begin to be expressed dorsally, and at stage 23 in the most dorsal part of the somites corresponding to the dermomyotome (Fig. 6A, st. 23). *Meox2* has already been reported as a marker of dermomyotome in *Xenopus* [23] and the double KO mice for the two *Meox* genes, *Meox1* and *2*, display drastic anomalies in all somitic derivatives, dermomyotome included with severely reduced pax3 expression [55]. To confirm that the *Meox2* expressing cells at stage 14 give rise to the dermomyotome, we realized cell tracing experiments by injecting fluorescent dyes at stage 13 in the most lateral part of presomitic mesoderm. The location of the fluorescent dye (red) at stage 18 in the lateral part of the somitic domain near the muscle fibers stained by 12/101 antibody corresponds to the *Meox2* expression domain (Fig 6B, stage 18). At stage 23, the comparison of the fates of the lateral cells (WGA-fluorescein/green) to the most lateral cells (WGA-rhodamine/red) indicated that the most lateral cells of presomitic mesoderm give rise to the most dorsal cells of the somites, corresponding to the dermomyotome (Fig 6B, stage 23). To support this view, we proceeded to ablation of the most lateral cells of presomitic mesoderm at stage 14, checked that *Meox2* expression was decreased at stage 19 (87%, n = 18) (Fig. 6E) and showed that absence of these cells dramatically affected dermomyotome formation evaluated by *Pax3* expression at the tailbud stage (87.8%, n = 33) (Fig. 6E). Neither the sham-operated embryos (73.7%, n = 19) (Fig. 6C), nor the ablation of less lateral presomitic mesoderm (85%, n = 20) (Fig. 6D) or lateral plate mesoderm (data not shown) leaded to a decrease in *Pax3* expression. These data confirm that dermomyotome comes from the most lateral cells of the presomitic mesoderm. By gain and loss of function experiments, we tested the effect of *Paraxis* and *Mef2d* on *Meox2* expression during neurulation. *Meox2* expression decreased in both *Mef2d* (68%, n = 50) and *Paraxis* (66.7%, n = 66) morphants (Fig. 7A). The phenotype was rescued by injection of Mef2dF (67.1%, n = 82) and ParaxisF’ (67.3%, n = 55) mRNA respectively (Fig. 7A). To test if *Meox2* could be a direct target gene of Paraxis and Mef2d, ParaxisGRF and Mef2dGRF were induced to translocate into nuclei by dexamethasone, after translation inhibition by cycloheximide. Induction of ParaxisGRF (71.1%, n = 45 without CHX, 69%, n = 58 with CHX) or MEF2GRF (61.2%, n = 55 without CHX, 61.2%, n = 62 with CHX) led to a moderate increase of *Meox2* expression, with or without cycloheximid treatment (Fig. 7B). The strongest effect was achieved when both (61.5%, n = 65 without CHX, 73.6%, n = 53 with CHX) were co-injected (Fig. 7B). These experiments suggest a combined action on *Meox2* promoter, which was verified by luciferase assays on the proximal promoter of *Xenopus tropicalis Meox2* gene, fused to a luciferase reporter (p*meox2*-luc). Luciferase activity of *pmeox2* was induced in COS7 cells transiently cotransfected with *Mef2d*, *Paraxis* or both (Fig. 7C). Combined transfections of *Mef2d* with *Paraxis* were required to get a maximal induction of luciferase activity, confirming the cooperative effect of Mef2d and Paraxis on *Meox2* expression (Fig. 7C). To test whether Mef2d could physically interact with Paraxis, co-immunoprecipitation assays were realized by using extracts from COS7 cells overexpressing a V5/His tagged full length *Mef2d* with either Gal4 alone or full length Paraxis fused to Gal4 (Fig. 7D). Mef2d-V5/His was immunoprecipitated with Ni-NTA beads, specific for the C-terminal His tag of Mef2d. Gal4-Paraxis was detected by immunoblotting against Gal4, indicating that Mef2d physically interacts with Paraxis (Fig. 7D).

### Meox2 Promotes Dermomyotome Formation Downstream of Paraxis and Mef2d

**Figure 8:**
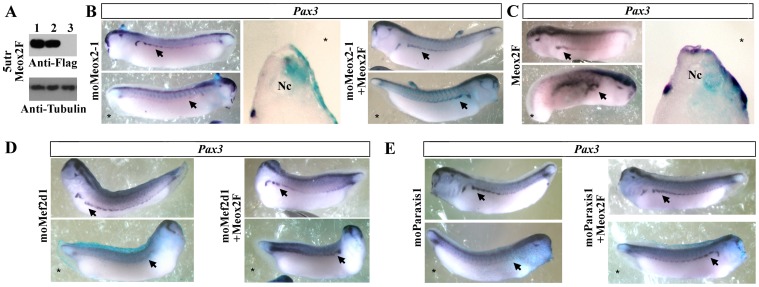
Meox2 acts downstream of Paraxis and Mef2d on dermomyotome formation. (A). Western blot with anti-flag and anti-tubulin antibodies of late gastrula embryos injected bilaterally either with 300 pg of 5utrMeox2F synthetic mRNA alone (lane1) or with oligomorpholinos: moControl (lane 2) or moMeox2-1 (lane3). (B) Embryos injected unilaterally with 20 ng of moMeox2-1 were submitted to *in situ* hybridization with *Pax3* antisense probe at the tailbud stage (lateral view or transverse section). A co-injection of Meox2F mRNA with moMeox2-1 was able to rescue the phenotype of moMeox*2*-1 (lateral view). (C) *Pax3* mRNA expression on lateral views and section of embryos at the tailbud stage after unilateral injection of Meox2F (200 pg). (D, E) Pax3 mRNA expression on lateral view of embryos at the tailbud stage after unilateral injection of moMef2d1(D) or moparaxis1 (E). Rescue experiments by Meox2F (100 pg) restored the phenotype of moMef2d1 (D) and moparaxis1 (E) morphants. Arrows indicate hypaxial expression of *Pax3*. (*) Injected side. Nc, notochord. For complete statistical data, see supporting information, figure S2.

The role of Meox2 on dermomyotome formation was investigated by gain and loss of function experiments. The efficiency of translation inhibition was demonstrated by injections of a blocking MO (moMeox2-1) and the mRNA coding for flag-tagged 5utrMeox2 (Fig. 8A). Unilateral injection of *Meox2* morpholinos (moMeox2-1) at the two cell-stage induced a decrease of *Pax3* expression (68.3%, n = 82) at stage 30 which was restored (68.9%, n = 61) in rescue experiments (Fig. 8B). Conversely, a *Meox2* injection at the two cell-stage induced an extension of *Pax3* (61.5%, n = 81) expression domain at stage 30 (Fig. 8C). As mentioned previously, the timing of *Mef2d* accumulation during *Xenopus* embryogenesis does not support a direct activation of *Pax3* gene in dermomyotome. Moreover, the lateral extension of *Pax3* expression domain observed after co-injection of *Paraxis* and *Mef2d* could be a consequence of the combined action of *Paraxis* and *Mef2d* on *Meox2* expression in the most lateral part of the presomitic mesoderm at the beginning of neurulation. So, we examined the hierarchical relationship existing between *Mef2d*/*Paraxis* and *Meox2* on *Pax3* expression. At stage 30, *Mef2d* (76.3%, n = 72) morphants (Fig. 8D) as well as *Paraxis* (76.2%, n = 84) morphants (Fig. 8E) exhibited a decrease of *Pax3* expression domain in the injected side that was restored (62.5%, n = 80 for Mef2d morphants, 67.6%, n = 68 for Paraxis morphants) by the injection of *Meox2* (Fig.8D, 8E). These data clearly demonstrate that *Meox2* acts downstream of *Paraxis* and *Mef2d* on *Pax3* expression during dermomyotome formation.

## Discussion

This report reveals a previously unrecognized regulatory network within myogenesis and points out an unexpected and pivotal role of one member of the Mef2 family, *Mef2d*, as a coupling factor of lateral myogenesis and dermomyotome formation in *Xenopus* embryos.

### Mef2d Acts as an Upstream Regulator of *Myod* Expression during Lateral Myogenesis in *Xenopus*

We have previously shown that during myotome formation, the medial myogenesis gives rise to the first differentiated fibers located near the notochord and the lateral myogenesis gives rise to dorsomedial and ventrolateral cell populations [6]. The lateral myogenesis is characterized by a myogenic program with strong *Myod* expression and without sustained *Myf5* expression [6]. Here, we showed by loss of function experiments, that *Myod* is necessary for lateral myogenesis as observed in zebrafish [5]. *Mef2d* expression extends in a large paraxial domain and precedes *Myod* expression in lateral presomitic mesoderm. By gain and loss of function experiments, we showed that *Mef2d* is required for *Myod* expression in lateral presomitic mesoderm. A Mef2 binding site has been indeed described on *Xenopus Myoda* promoter, and was shown as necessary for *Myod* expression in cell culture [43], [44]. However, in our experiments, *Mef2d* alone is not able to induce ectopic expression of Myod, which means that other factors probably participate to the *Mef2d* transactivation activity on the *Myod* promoter. Moreover, *Mef2d* morphants seem essentially affected in the hypaxial region of the myotome and express normal level of *Myogenin* in the epaxial region during the second myogenic wave (data not shown), suggesting that signals emanating from the neural tube and the dorsal ectoderm might be sufficient for the development of the epaxial region.

The function of Mef2d seems not conserved in anamniote, as Mef2 proteins are expressed after *Myod* and is not involved in the control of *Myod* expression during lateral myogenesis in zebrafish [56]. The early activity of Mef2 is actually similar in *Drosophila* and *Xenopus*, as *Drosophila Mef2* regulates several steps of muscle differentiation, from regulation of muscle identity genes to terminal muscle differentiation process [57], [58]. It should also be noted that a recent work [24] showed that Mef2 protein cooperates with the coactivator Mastr to regulate the transcription of *Myod* during mouse skeletal muscle regeneration. Mastr is also expressed in the *Xenopus* lateral presomitic mesoderm [59]. Although the function of Mef2 protein seems not conserved during embryonic myogenesis between *Xenopus,* zebrafish and mouse, at least part of this genetic circuitry has been used in mammals in the context of muscle regeneration.

Fgf plays various roles during early development of *Xenopus*, including the development of dorsal mesodermal structures [60]. In particular, Colas et al. [50] have described an inhibitory effect of Fgf on the development of intermediate mesoderm to the benefit of muscle development, during gastrulation. Moreover, Fgf can induce *Myod* expression in cap animal assays [61] or during development [31] either directly [61] or indirectly [62]. In zebrafish, lateral Myod-dependent myogenesis [5] develops in an Fgf8 dependent way. In *Xenopus*, gain and loss of function experiments showed that Fgf8b modulates *Myod* expression in the lateral paraxial mesoderm essentially [31], suggesting that Fgf8 constitutes an important signal for lateral myogenesis. The treatment of *Xenopus* embryos with the inhibitory drug SU5402 from stage 11.5/12 (this work) suggests that the Fgf effect could continue until the beginning of neurulation where *Fgf*8 is expressed in the posterolateral region [31]. While *Myod* is clearly expressed in the lateral marginal zone at the beginning of gastrulation [40] and could be directly activated by Fgf, its expression seems to decrease afterwards in this region. In this report, we showed that the later Fgf effect on lateral *Myod* expression is mediated by Mef2d, as suggested by the appearance of Mef2 promoter-binding activity in the beginning of neurulation [44]. Morever, two important works from Harland laboratory [40], [41] are consistent with this shift in *Myod* gene regulation at the gastrulation-neurulation transition. First, the initial expression of *Myod* in the ventrolateral domain in gastrula is not sufficient to give rise to muscle tissue in culture [40] suggesting that the initial *Myod* expression is transient in this region. Second, *Myod* gene is the target of a new inductive signal from neural plate during gastrulation, beginning probably at the end of gastrulation and pursuing during early neurulation [41]. Indeed, Mef2d seems to be a good candidate to be the relay of the neural plate signal in mesoderm, but the relationship between the neural plate signal on one hand, Mef2d and Fgf8 on the other hand, needs to be determined. Finally, other transcription factors could be involved, such as Egr1 that controls *Myod* expression in an Fgf dependent way [62]. The relation between Egr1 and Mef2d in lateral myogenesis remains also to be established.

### Identification of Dermomyotome Precursors in *Xenopus*

**Figure 9:**
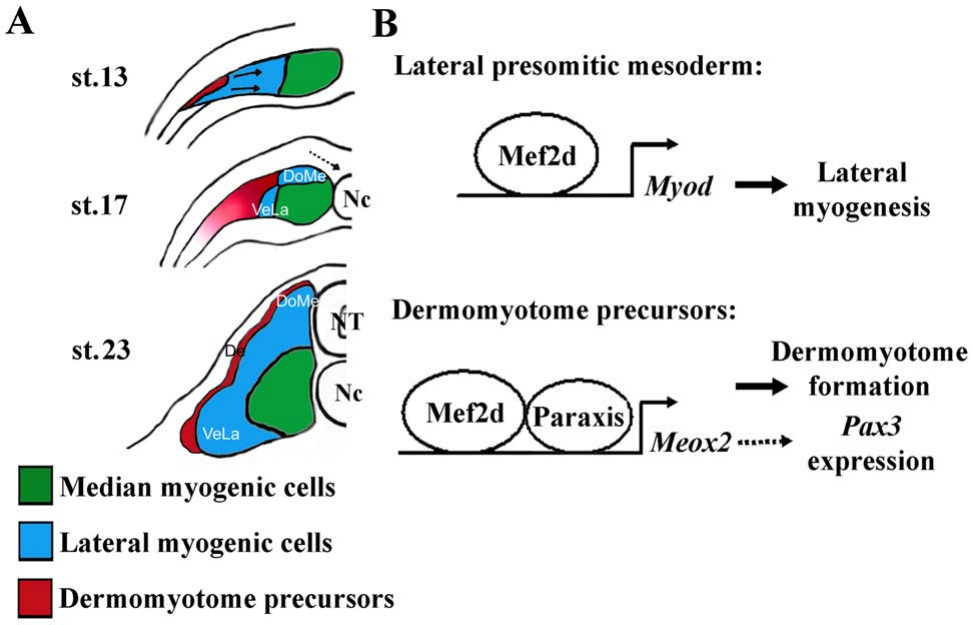
Schematic representation of myotome and dermomyotome formation in *Xenopus*. (A) The most lateral *Meox2* expressing cells of the paraxial mesoderm give rise to the dermomyotome (De) whereas the lateral myogenic cells give rise to a dorsomedial (DoMe) and a ventrolateral (VeLa) cell population. The medial myogenic cells differentiate first and remain associated with the notochord [5]. Arrows design the movement of lateral paraxial mesoderm. Dotted arrows design the movement of invagination of neurectodermal cells. NT, neural tube. Nc, notochord. (B) Mef2d couples lateral myogenesis to dermomyotome formation: In lateral presomitic cells, Mef2d transactivates the *Myod* gene and in the most lateral presomitic cells Mef2d and Paraxis transactivates the *Meox2* gene.

Even if we cannot totally exclude that some cells ouside of the presomitic mesoderm could be mixed with presomitic mesoderm to participate in dermomyotome construction, altogether, gene expression data, cell lineage tracing, ablation and gain-and-loss of function experiments strongly suggest that dermomyotome precursors are localized in the most lateral part of the presomitic mesoderm (Fig. 9A). To support our data, it has already been shown in *Xenopus* that satellite cells originate from lateral mesoderm [63] but the authors concluded that they originate “from the dorsolateral plate rather than from the paraxial mesoderm”. Considering that satellite cells come from the dermomyotome in amniotes [64], [65] and in the light of our results showing lateral expression of *Meox2*, it could be proposed that most satellite cells originate from dermomyotome precursors rather than from dorsolateral plate mesoderm. Concerning the epaxial region of somites, its development seems independent from the formation of lateral dermomyotome precursors since it develops almost normally in *Mef2d* morphants. The compartmentalization of the presomitic and somitic mesoderm in *Xenopus* presents some characteristics that could impose specific constraints and explain the existence of dermomyotome precursors. First, it is well known that paraxial mesoderm, notochord and neural tube are subjected to extension convergent movements during neurulation leading to an anteroposterior elongation of the embryo [66]–[68]. These extension convergent movements are particularly strong in paraxial mesoderm. As cells are specified early, it seems necessary to couple cellular movements to the cellular fate to have the right tissue at the right place. Second, the presomitic mesoderm and the newly formed somites are not naive tissue due to the presence of specified and differentiated muscle cells. In this context, some cells of the somites may be protected from myogenic signals to remain competent to form the dermomyotome. Would *Meox* be able to maintain the competence of these cells? In this case, since the two *Meox* genes are expressed in the entire naive somites of amniotes [55], [69] and are involved in dermomyotome and sclerotome formation [55], the function of *Meox* genes could be similar in amniotes and anamiotes.

### Mef2d Couples Dermomyotome Formation and Lateral Myogenesis in *Xenopus*

In amniote myogenesis, Paraxis is necessary for the development of the hypaxial region of the somites [53] and has been involved in the regulation of somite epithelialization and the maintenance of the epithelial state of dermomyotome [70]. Here, we identified a new and early function of Paraxis in activating *Meox2* expression in the dermomyotome progenitors which develop in association with lateral myogenic cells in *Xenopus* (Fig. 9A). Mef2d is also involved in dermomyotome formation. It co-localizes with *Paraxis* in the most lateral part of presomitic mesoderm, interacts with Paraxis by co-immunoprecipitation assay and cooperates with Paraxis either to enhance *Meox2* expression in the dermomyotome progenitors or to activate *Xenopus tropicalis Meox2* promoter in luciferase assays. Moreover, an evolutionary conserved Mef2 binding site on the mouse *Meox2* promoter is necessary for *Meox2* expression in cultured cells [34]. However, the effect of Mef2d alone is relatively moderate and is time-limited to the beginning of neurulation since *Mef2d* is not coexpressed with *Meox2* afterwards. Interaction of Mef2d with histone modifying enzymes that introduce epigenetic modifications [71] without interacting directly with the RNA polymerase II complex could explain this transitory role of Mef2d.

Finally, Mef2d can directly regulate muscle identity genes (Fig. 9B). Mef2d function in coupling lateral myogenesis to dermomyotome formation does not seem to be conserved during evolution. This is not the case in zebrafish, another anamniote species, where Mef2d is not required for lateral myogenesis [5], [56] nor for dermomyotome formation. The compartmentalization of somites presents some differences between *Xenopus* and zebrafish. In zebrafish, lateral myogenesis and dermomyotome formation take place after somitogenesis. Their positioning results from the coordinated rotation of two somitic populations: the anterior one expressing *Pax7* gives rise to the most exterior dermomyotome and the posterior one expressing *Myod* gives rise to the more interior fast lateral fibers [72]. In this case, the early coupling function of Mef2 protein in presomitic mesoderm might have been lost. In mammals, it is well established that trunk and limb muscles derive from the dermomyotome [9], which formation is the initial event of myogenesis, whereas in *Xenopus*, the first medial and lateral myogenesis appears before the dermomyotome. *Mef2d*-null mice are viable and present no obvious skeletal muscle development defects [18], [20]. Although we cannot exclude that the function of Mef2d could be compensated by other members of the Mef2 family, early coupling function of Mef2d has probably been lost during evolution since the dermomyotome precursors are no longer associated with lateral myogenesis during their formation. In mammals, Mef2 proteins are able to enhance transcription of the transfected *Meox2* promoter, particularly in C2C12 myoblasts [34], therefore we cannot exclude that Mef2 might control *Meox2* expression in one of the mouse myogenesis.

### Similarities between Dermomyotome and Tendon Formation in *Xenopus*

Amazingly, we observed striking similarities between dermomyotome and tendon formation in *Xenopus*. Both are associated with myogenic tissue during their formation and both express a member of the Mef2 family and one of the bHLH Twist family: *Mef2d* and *Paraxis* in the progenitors of dermomyotome, *Mef2c* and *Scx* in those of larval tendon [32]. Moreover, the *Paraxis* and *Scx* genes of jaw vertebrates result from the duplication of a unique gene named *Parascleraxis* in the jawless vertebrate lamprey [73], supporting the existence of similar function of Scx and Paraxis which could be linked to their expression to muscle-associated tissue as it is the case in *Xenopus*. From these shared characteristics, we hypothesize that a vertebrate ancestral mechanism might have coupled the development of myogenic cells to the formation of associated tissues during somite compartmentalization. In the cephalochordate amphoxius, somitic muscles develop, but no dermomyotome has been observed [74]. In the jawless fish lamprey, lateral cells of somites are equivalent to dermomyotome [74] and expression of the *Pax3/7* gene [65] is similar to that of *Pax3* in *Xenopus*. In a common vertebrate ancestor, muscle segmented cells might have played a scaffolding role in the development of the dermomyotome.

In conclusion, we identified a new genetic regulatory network involved in myogenesis during *Xenopus* embryogenesis and it is important to know to what extent it is used in one of the amniote myogenesis. In a more general way and beyond somite development, the significance of the cooperative effect between the members of the Twist [75] and the Mef2 family [76] should be addressed since both are involved in the development of other tissues, such as heart and neural crests.

## Supporting Information

Figure S1 **Sequences targeted by oligomorpholinos.** 5′utr sequence of the two genes coding for Myod, Myf5, Mrf4, Mef2d, Paraxis and Meox2 with sequences recognized by oligomorpholinos.
(DOCX)

Figure S2 **Statistical analysis of injected or treated embryos.** Histograms showing percentage of embryos with enhanced, decreased or unchanged expression.
(DOCX)

Table S1 **Primers used for cloning of in situ hybridization probes.** Forward and reverse primers used for the cloning of Paraxis, Meox2, Pax7 and Xbra probes.
(DOCX)
