# Supplementary material for: Mef2d Acts Upstream of Muscle Identity Genes and Couples Lateral Myogenesis to Dermomyotome Formation in Xenopus laevis
Source: PLoS One. 2012 Dec 31;7(12):e52359. doi: 10.1371/journal.pone.0052359 (PMC3534117; doi:10.1371/journal.pone.0052359)
Supplement: Figure S1 — Sequences targeted by oligomorpholinos. 5′utr sequence of the two genes coding for Myod, Myf5, Mrf4, Mef2d, Paraxis and Meox2 with sequences recognized by oligomorpholinos. (DOCX) [file pone.0052359.s001.docx]

**Figure S1.** **Sequences targeted by oligomorpholinos.**

5’utr sequence of the two genes a and b coding for Myod, Myf5, Mrf4, Mef2d, Paraxis and Meox2. Initiation codon ATG in yellow. Sequences targeted by oligomorpholinos in green.

Myod:

a ----------------CGGCTGTTTCTATGGAGCTCTTGCCCCCAGCACT------CAGG

| | | | | | | | | | | | | | | | | | | | | | | | | | | | | | | | | |

b TGGGAGTAGTGACTTTGCGCTGTTTCTATGGAGCTGTTGCCCCCACCACTGCGGGACATG

a ----------------CGGCTGTTTCTATGGAGCTCTTGCCCCCAGCACT------CAGG moMyod1a

| | | | | | | | | | | | | | | | | | | | | | | | | | | | | | | | | |

b TGGGAGTAGTGACTTTGCGCTGTTTCTATGGAGCTGTTGCCCCCACCACTGCGGGACATG moMyod1b

Myoda:

1 gcaactgccacaagtcaagtctctgctccaaactgcgggtatttggcctggacccccgag moMyoD2a

61 ttgtggatctgtgggaccccccggctgtttctatggagctcttgcccccagcactcaggg

Myodb:

1 atcaggtgccctgagagggtgagagctgccaagagtccagatttcctacaacggggcaag

61 gtgactgtgcgtgggcattcggactggaccccagagctgtggatcttttaaaccttcgct moMyoD2b

121 gggagtagtgactttgcgctgtttctatggagctgttgcccccaccactgcgggacatgg

Myf5:

a 1 AGAAAGCCAACCAAAGGAACTCCTAAGAGATTTCCTGAAACCTGATTGCTTCAACTCCAC 60 moMyf5a

||||||| ||||||||| | || | | ||||||| | |||||||||||| ||||||||

b 1 AGAAAGCGAACCAAAGG-A--GCT-A-A-ATTTCCTAAGACCTGATTGCTTGAACTCCAC 54

a 61 TGAGCATCTTTCTAAGCAGCACTATTCAGAATGGAGATGGTAGATAGCTGCCATTTTTCC 120

||| ||||||||||| |||||||| |||||||||||||||||||||||||||||||||||

b 55 TGAACATCTTTCTAAACAGCACTAGTCAGAATGGAGATGGTAGATAGCTGCCATTTTTCC 114 moMyf5b

Mrf4:

a 1 ACTACTTAAATTCCTTCAGGGCTGTGCTGTTTTGAGCACGCCACTTAGCTAAGCTGCCAG 60 moMrf4-2

||||||||||||||||||||||||||||||||||||||||||||||||||| ||||||||

b 1 ACTACTTAAATTCCTTCAGGGCTGTGCTGTTTTGAGCACGCCACTTAGCTAGGCTGCCAG 60

a 61 AGCAATCAAGGGCAAAGCATAATGATGGACCTATTTGAAACAAATTCCTATTTCTTTTAC 120

||||||||||||||||||||||||||||||||||||||||||||||||||||| ||||||

b 61 AGCAATCAAGGGCAAAGCATAATGATGGACCTATTTGAAACAAATTCCTATTTTTTTTAC 120

a 1 ACTACTTAAATTCCTTCAGGGCTGTGCTGTTTTGAGCACGCCACTTAGCTAAGCTGCCAG 60

||||||||||||||||||||||||||||||||||||||||||||||||||| ||||||||

b 1 ACTACTTAAATTCCTTCAGGGCTGTGCTGTTTTGAGCACGCCACTTAGCTAGGCTGCCAG 60

a 61 AGCAATCAAGGGCAAAGCATAATGATGGACCTATTTGAAACAAATTCCTATTTCTTTTAC 120 moMfr4-1

||||||||||||||||||||||||||||||||||||||||||||||||||||| ||||||

b 61 AGCAATCAAGGGCAAAGCATAATGATGGACCTATTTGAAACAAATTCCTATTTTTTTTAC 120

Mef2d :

a 37 TGAAGCCGGCCGAAAGCTTTCAGAACATCTGGAACCAGCAGGAGAAGAACATGAACTGAA 96

|||||||||||||||||||||||||||||||||||||||||||||| ||||||||||||

b 159 TGAAGCCGGCCGAAAGCTTTCAGAACATCTGGAACCAGCAGGAGAATAACATGAACTGAC 218 moMef2d2

a 97 GAGGAAAGAAAAAAAAAGGGGGTTCG-TTTTCAATTATTTCATGAACACATTGTGAGGAG 155

| || | |||||||||| ||| |||||||||||||||||||||||||||||| ||

b 219 G-GG---G----AAAAAGGGGGCTCGTTTTTCAATTATTTCATGAACACATTGTGAGAAG 270

a 156 CAAGAGGAGGGGGGTCAAAAAAAAACGGGAACCCGTTCACTGCAAGGTCTTCGCTGGTCG 215 moMef2d1a

|| ||||||||||||| |||||| ||||||||||||| | ||||||||| ||||||||

b 271 CAGGAGGAGGGGGGTC--AAAAAACCGGGAACCCGTTCGCCGCAAGGTCTCCGCTGGTCA 328 moMef2d1b

a 216 GTGAGGATTTTCCAGGAATCATGGGCAGAAAAAAGATCCAGATTCAGAGGATCACAGATG 275

|||||||||||||||||| |||||| ||||||||||||||||||||||||||||||||||

b 329 GTGAGGATTTTCCAGGAACCATGGGGAGAAAAAAGATCCAGATTCAGAGGATCACAGATG 388

Paraxis :

a 1 AGAAATAAATGCCCCGTTGGAGCTGAGAGGAA-T-AGGAGTTGAGTTGGCCCAGGGCTCA 58

|||| ||||| ||| ||||||||||||||||| | ||||| ||||| ||| ||| | ||

b 17 AGAACTAAATACCCTGTTGGAGCTGAGAGGAATTAAGGAG-TGAGTCAGCCTAGGACCCA 75 moParaxis1

a 59 CATGGCCTTCACCATGATCCGTTCCATGTCAACGCATGTGATTTATCCGGACATGTCCAT 118

|||||||||||||||||||||||||||| ||||||||| |||||| ||||| |||||||

b 76 CATGGCCTTCACCATGATCCGTTCCATGCCAACGCATGGGATTTACCCGGATGTGTCCAT 135

a 1 AGAAATAAATGCCCCGTTGGAGCTGAGAGGAA-T-AGGAGTTGAGTTGGCCCAGGGCTCA 58 moParaxis2a

|||| ||||| ||| ||||||||||||||||| | ||||| ||||| ||| ||| | ||

b 17 AGAACTAAATACCCTGTTGGAGCTGAGAGGAATTAAGGAG-TGAGTCAGCCTAGGACCCA 75 moParaxis2b

a 59 CATGGCCTTCACCATGATCCGTTCCATGTCAACGCATGTGATTTATCCGGACATGTCCAT 118

|||||||||||||||||||||||||||| ||||||||| |||||| ||||| |||||||

b 76 CATGGCCTTCACCATGATCCGTTCCATGCCAACGCATGGGATTTACCCGGATGTGTCCAT 135

Meox2:

a 40 CCACCAGGCTGAAGACCTGGATTATCTGATCTGTACTGAAAGTGTGCCGTTACTGTTTGG 99

||||||||||||||||||||||||||||||||||||||||||||||||||||||||||||

b 1 CCACCAGGCTGAAGACCTGGATTATCTGATCTGTACTGAAAGTGTGCCGTTACTGTTTGG 60

a 100 GAAAAATATCATTTTACAGTAGAAA-GTGACAGCGTTGTGGAGTTTAATCCTGCCAGAAT 158

|||||||||||||||||||| |||| ||||||||||||||||||||||||||||||||||

b 61 GAAAAATATCATTTTACAGT-GAAAAGTGACAGCGTTGTGGAGTTTAATCCTGCCAGAAT 119

a 159 CTCAAGCCTGAAACTTGAAACTTGCATGCCATG 191 moMeox2-1

|||||||||| ||||||||||||||||||||||

b 120 CTCAAGCCTGCAACTTGAAACTTGCATGCCATG 152

a 40 CCACCAGGCTGAAGACCTGGATTATCTGATCTGTACTGAAAGTGTGCCGTTACTGTTTGG 99

||||||||||||||||||||||||||||||||||||||||||||||||||||||||||||

b 1 CCACCAGGCTGAAGACCTGGATTATCTGATCTGTACTGAAAGTGTGCCGTTACTGTTTGG 60

a 100 GAAAAATATCATTTTACAGTAGAAA-GTGACAGCGTTGTGGAGTTTAATCCTGCCAGAAT 158

|||||||||||||||||||| |||| ||||||||||||||||||||||||||||||||||

b 61 GAAAAATATCATTTTACAGT-GAAAAGTGACAGCGTTGTGGAGTTTAATCCTGCCAGAAT 119

a 159 CTCAAGCCTGAAACTTGAAACTTGCATGCCATGGAACACACACTATTTGGC 191 moMeox2-2

|||||||||| ||||||||||||||||||||||||||||||||||||||||

b 120 CTCAAGCCTGCAACTTGAAACTTGCATGCCATGGAACACACACTATTTGGC 152
