# Supplementary material for: Mef2d Acts Upstream of Muscle Identity Genes and Couples Lateral Myogenesis to Dermomyotome Formation in Xenopus laevis
Source: PLoS One. 2012 Dec 31;7(12):e52359. doi: 10.1371/journal.pone.0052359 (PMC3534117; doi:10.1371/journal.pone.0052359)
Supplement: Figure S2 — Statistical analysis of injected or treated embryos. Histograms showing percentage of embryos with enhanced, decreased or unchanged expression. (DOCX) [file pone.0052359.s002.docx]

**Figure S2: Statistical analysis of injected or treated embryos.**

**Corresponding to Fig. 1:**


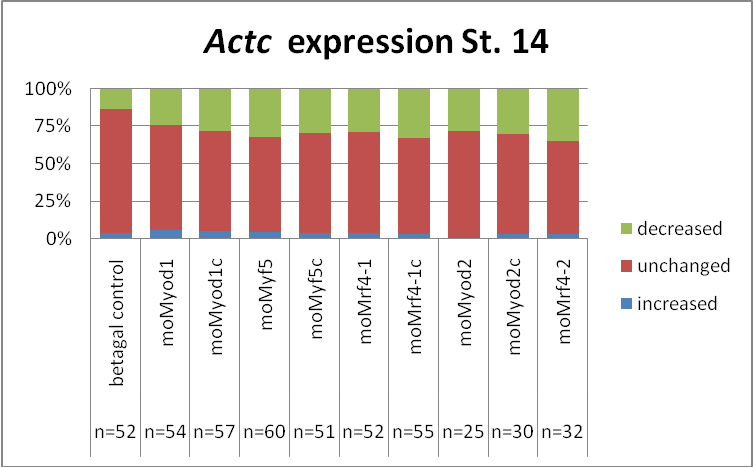

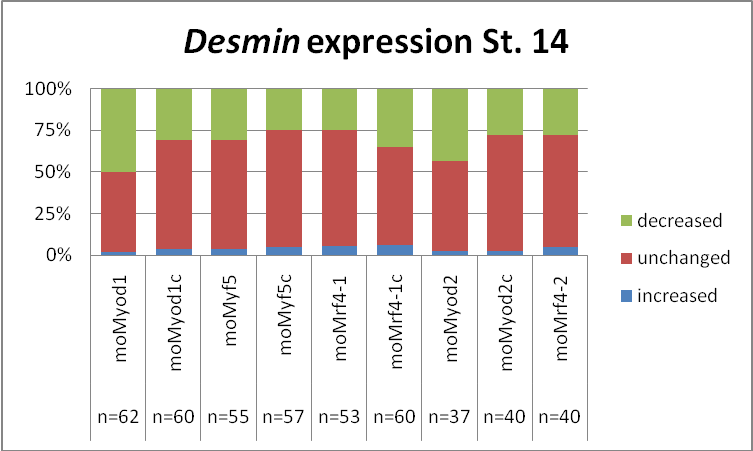

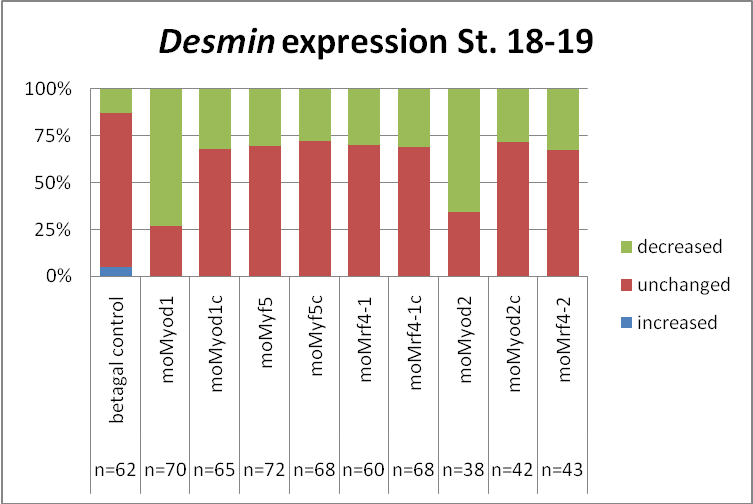

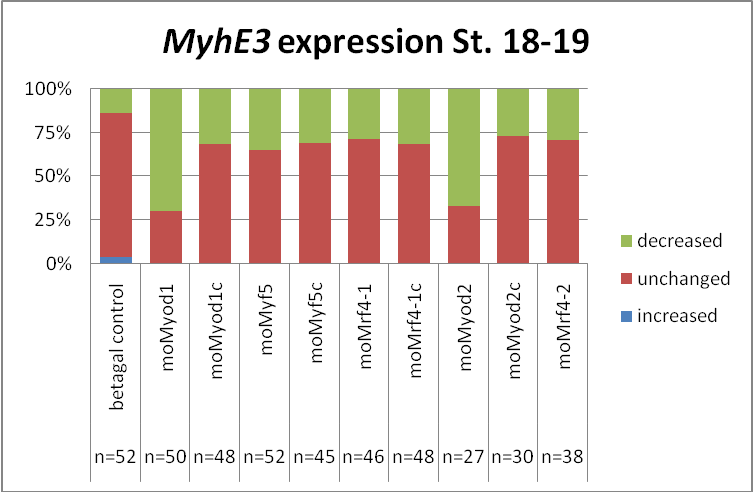

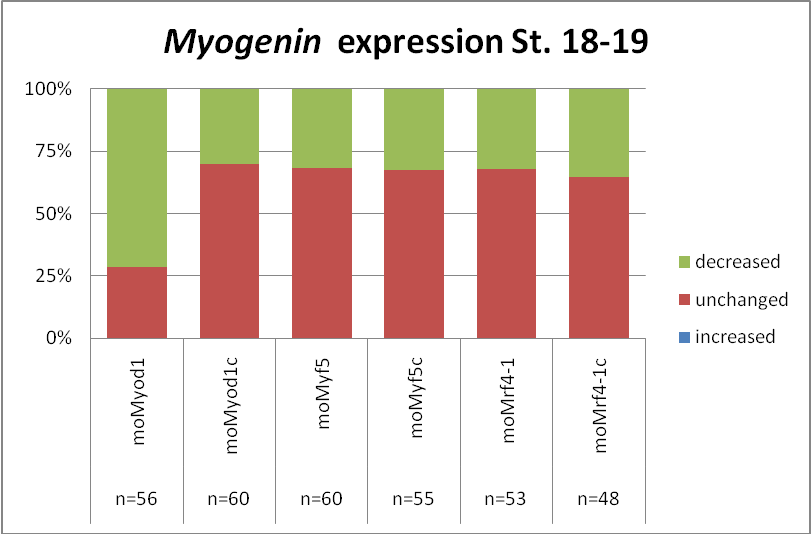

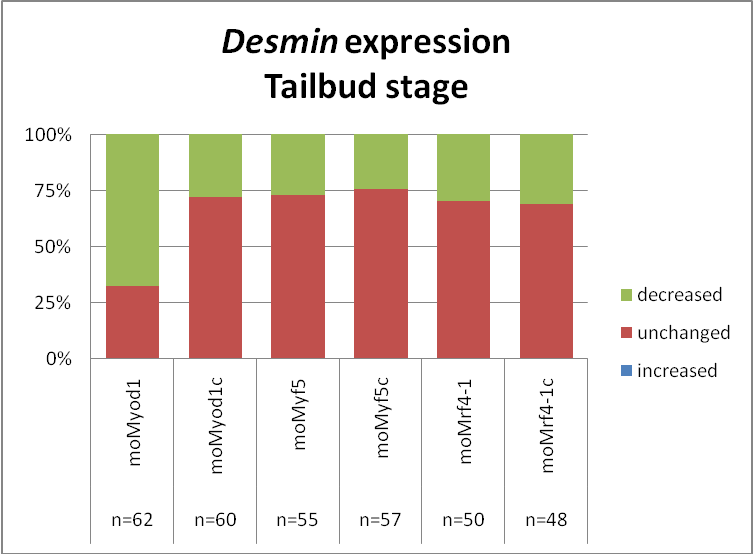


**Corresponding to Fig. 2:**


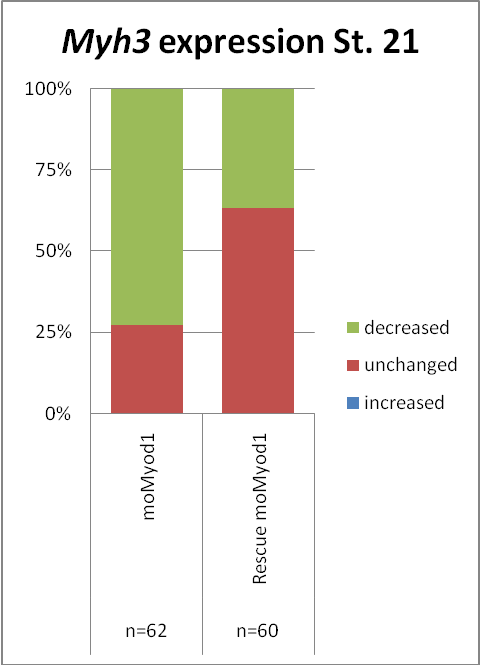

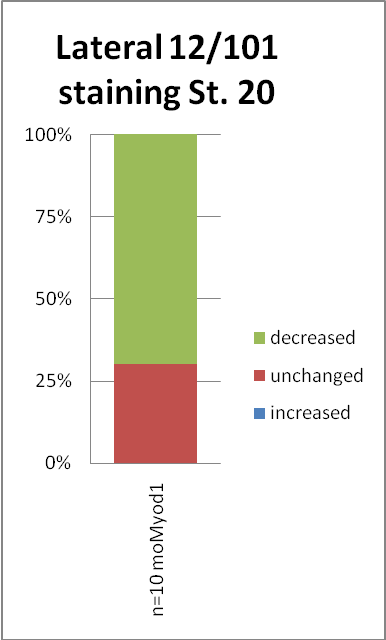

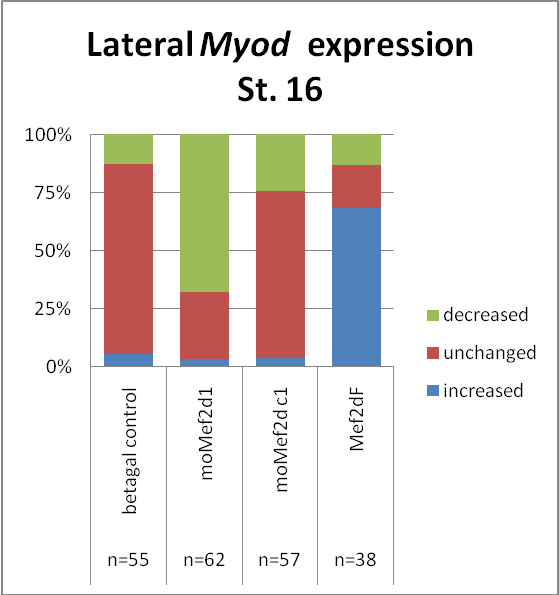

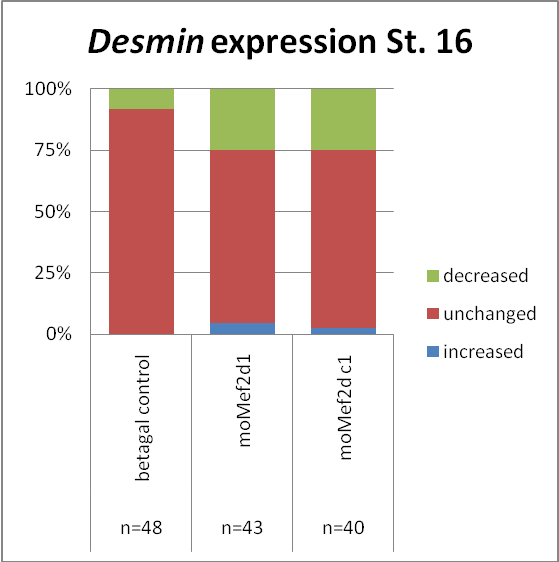

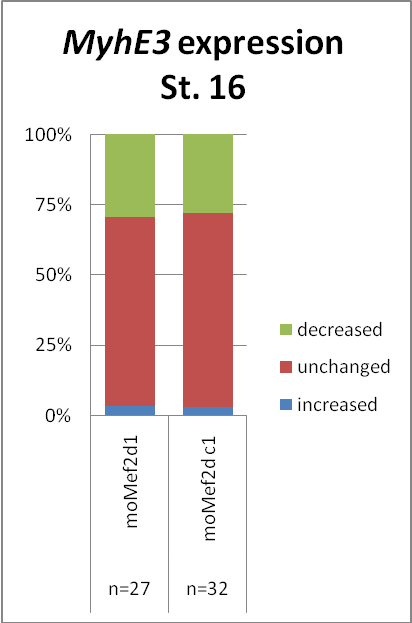

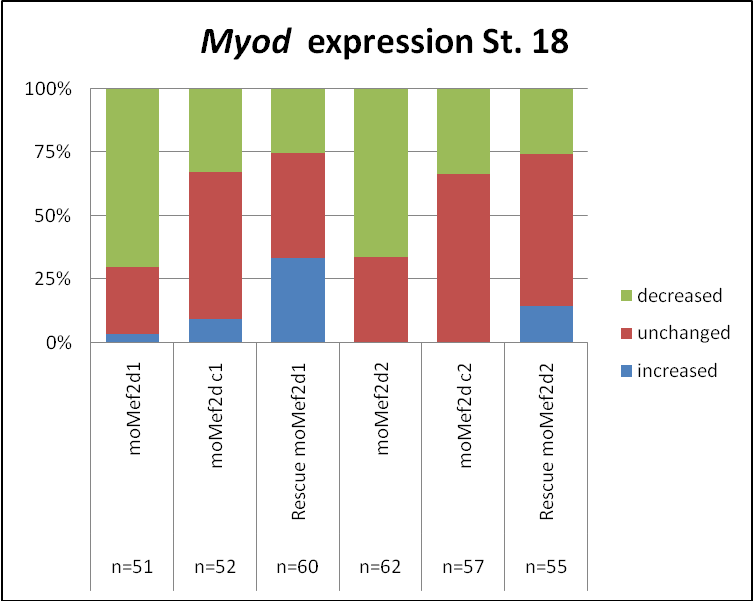

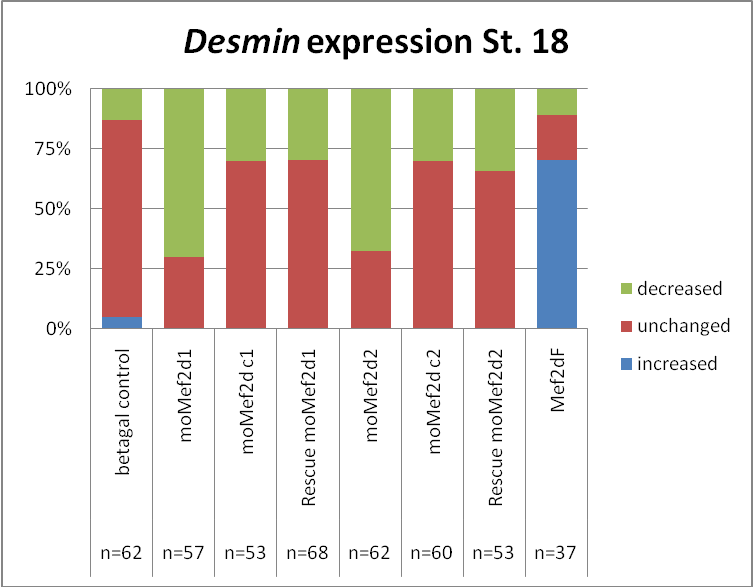

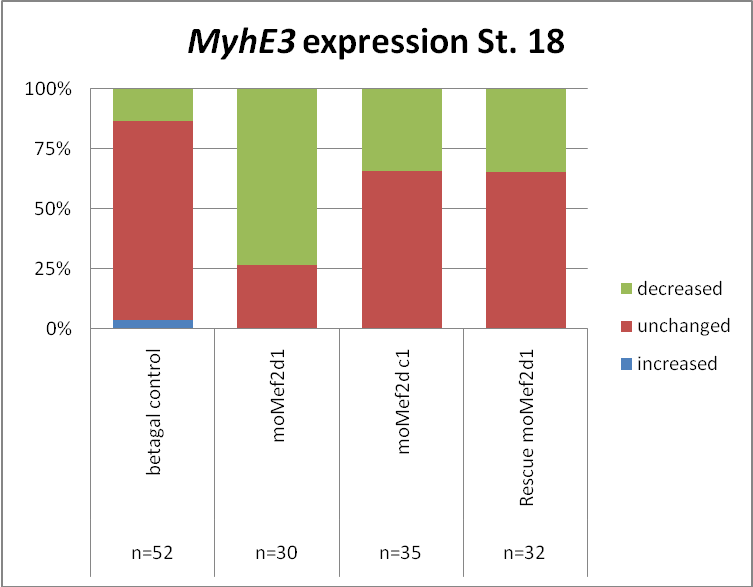

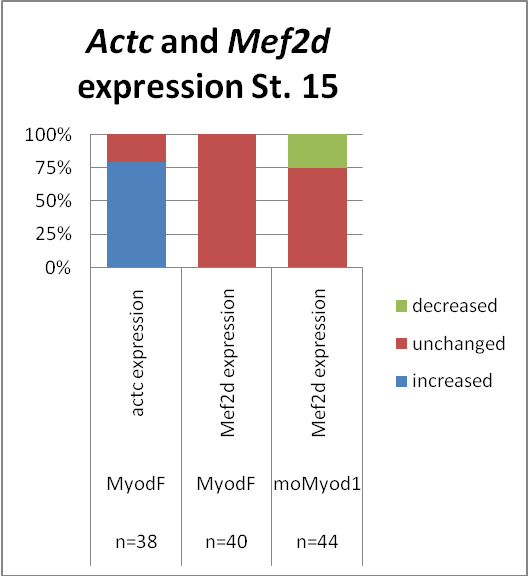


**Corresponding to Fig. 3:**


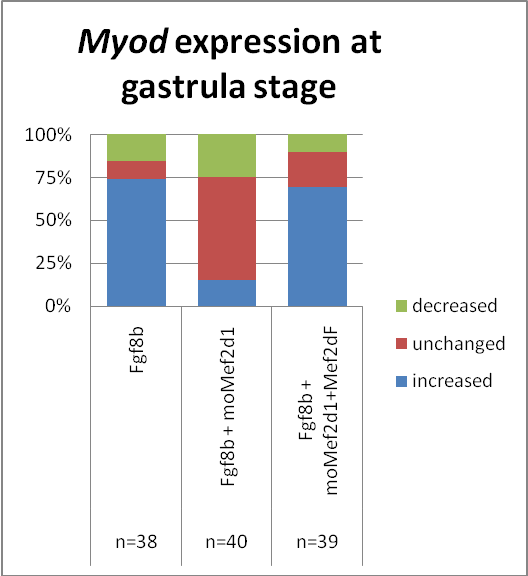

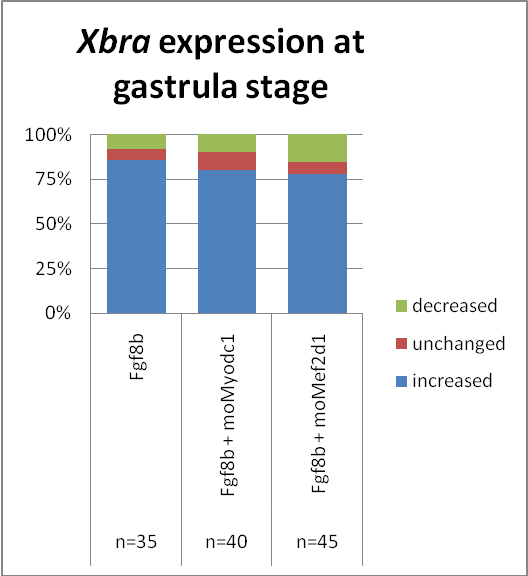

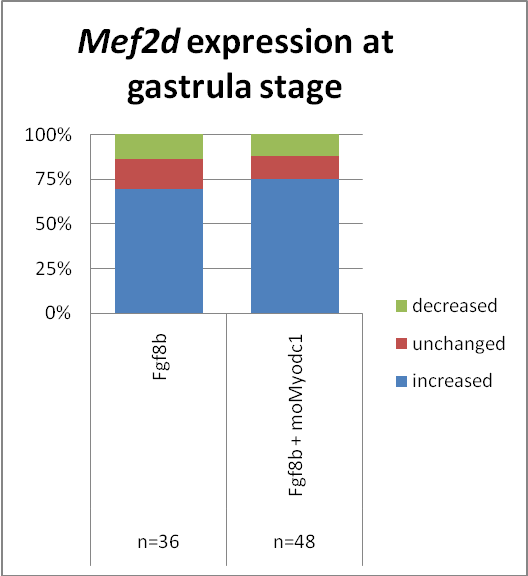

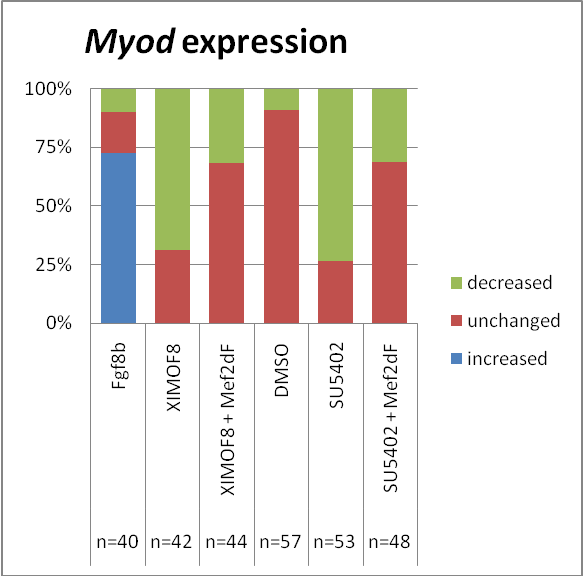

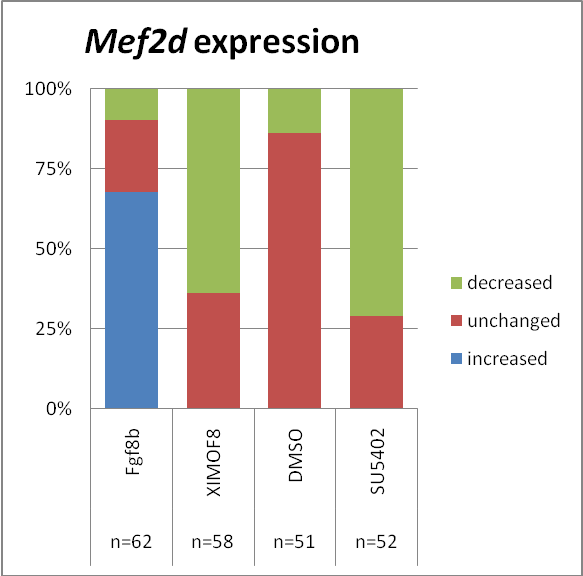


**Corresponding to Fig. 4:**


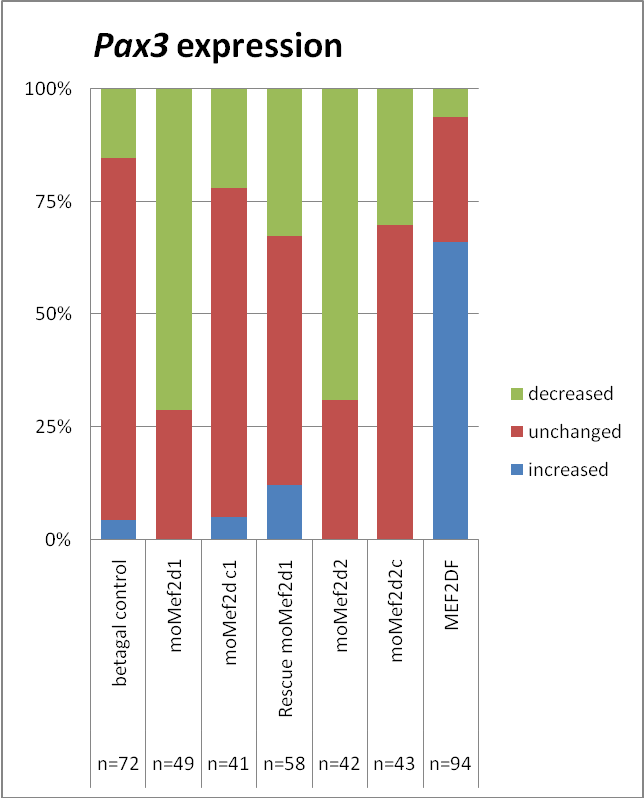


**Corresponding to Fig. 5:**


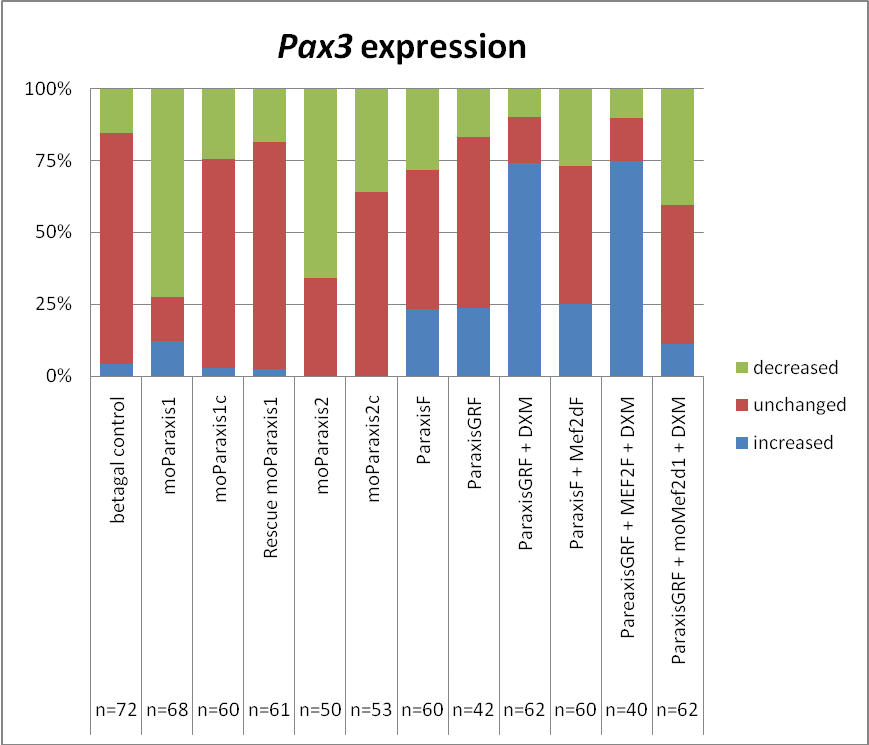


**Corresponding to Fig. 6:**


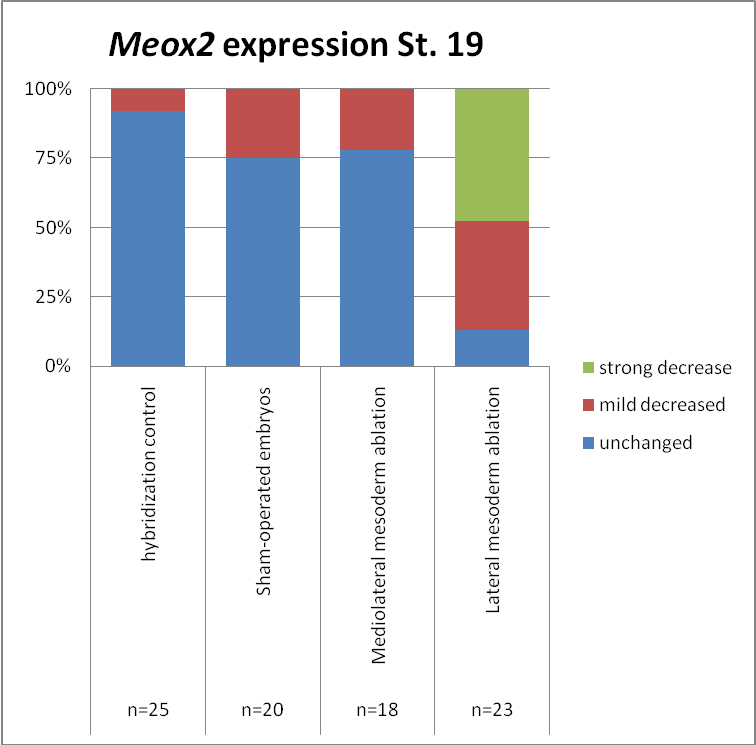


**Corresponding to Fig. 7:**


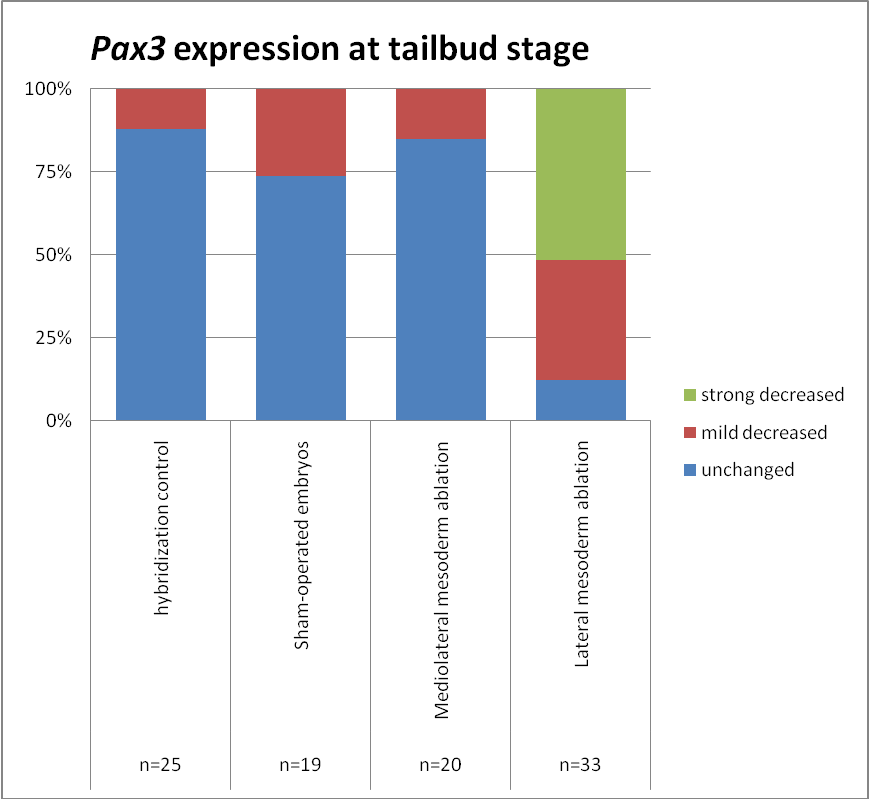

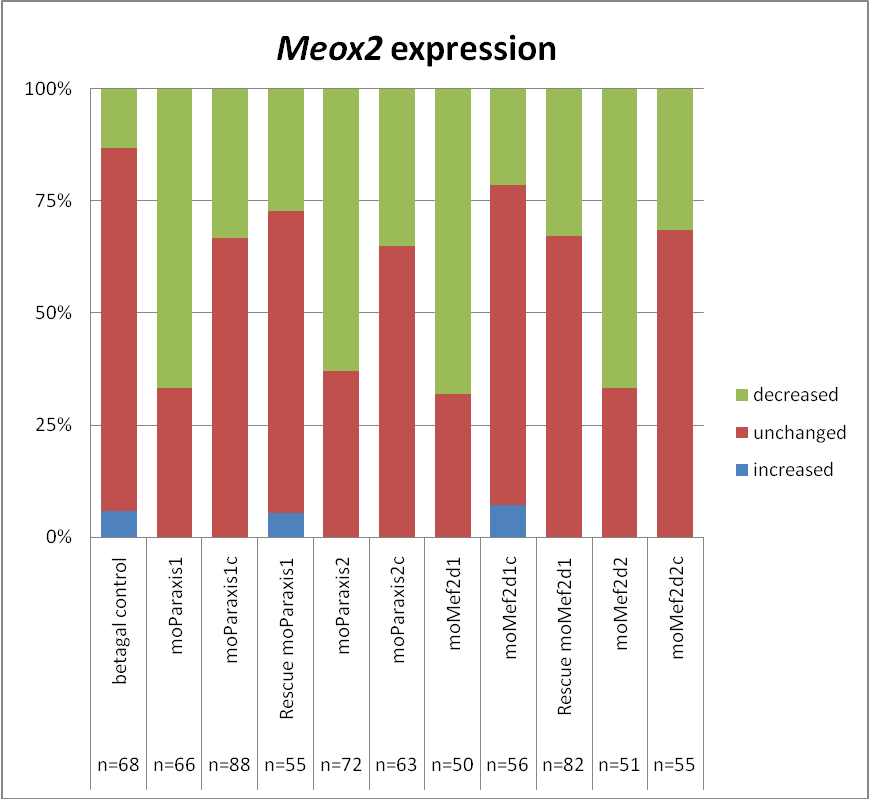

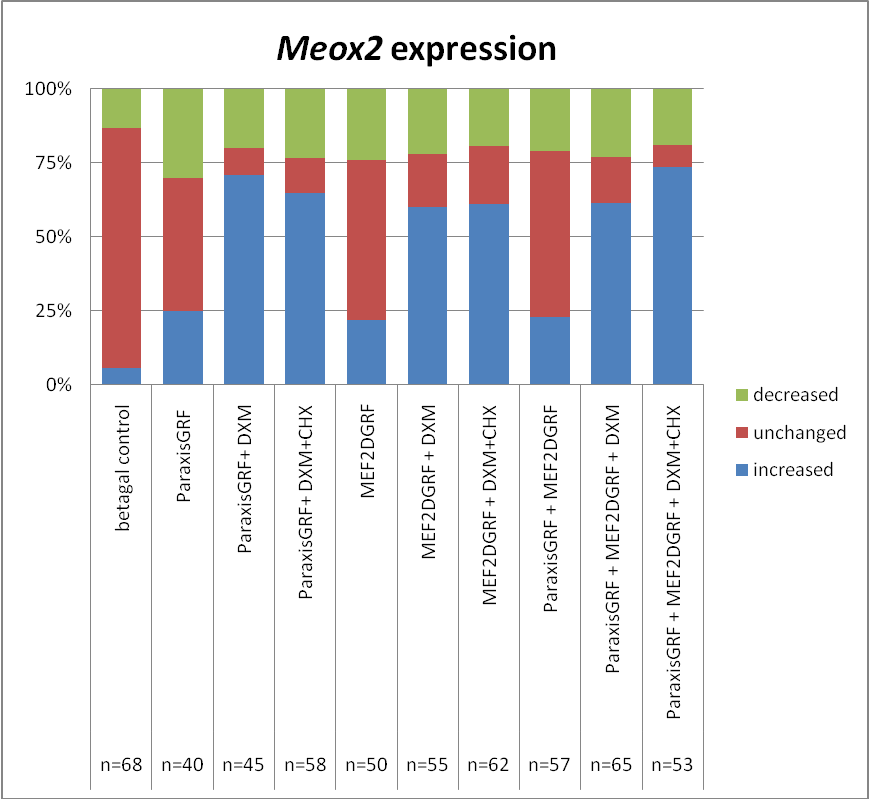


**Corresponding to Fig. 8:**


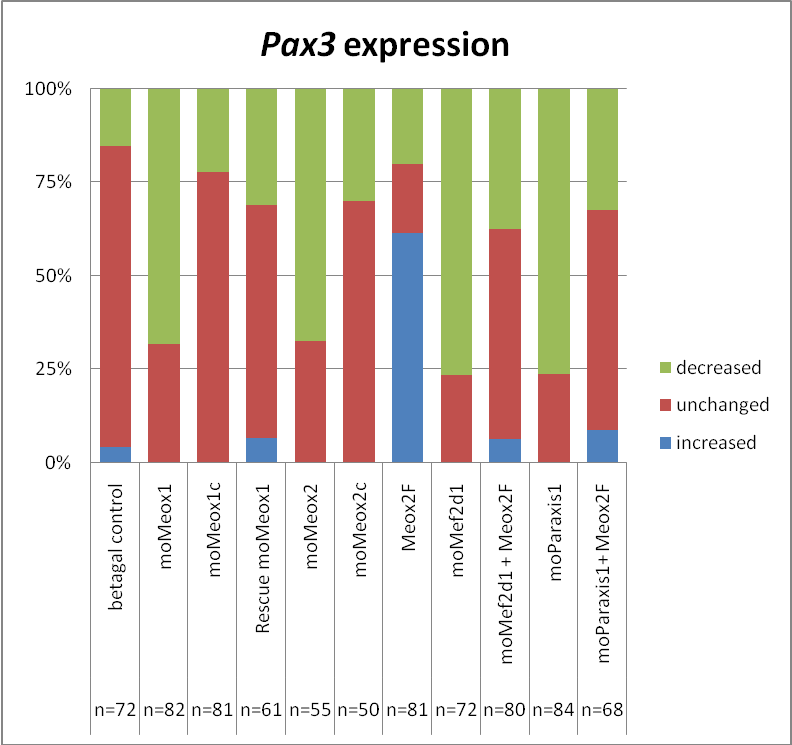


**Total number of embryos: n**

**Betagal control: embryos injected only with synthetic mRNA coding for betagalactosidase**
