# Supplementary material for: Mef2d Acts Upstream of Muscle Identity Genes and Couples Lateral Myogenesis to Dermomyotome Formation in Xenopus laevis
Source: PLoS One. 2012 Dec 31;7(12):e52359. doi: 10.1371/journal.pone.0052359 (PMC3534117; doi:10.1371/journal.pone.0052359)
Supplement: Table S1 — Primers used for cloning of in situ hybridization probes. Forward and reverse primers used for the cloning of Paraxis, Meox2, Pax7 and Xbra probes. (DOCX) [file pone.0052359.s003.docx]

**Table S1: Primers used for cloning of in situ hybridization probes**

|  | Forward primers | Reverse primers |
| --- | --- | --- |
| Paraxis | 5’-gccccgttggagctgagagg-3’ | 5’-GTGCCCACCAGACCTTGGTA-3’ |
| Meox2 | 5’-tgctcaccacaactacctga-3’ | 5’-GCAAGTATACCTGCCCATAG-3’ |
| Pax7 | 5’-gaaagcctttgagaggacac-3’ | 5’-CAGTTTTTCACCAAGTGGCA-3’ |
| Xbra | 5’-taaggaaccaccgctcagct-3’ | 5’-GTCTGTAGCAGCAGTCCCAA-3’ |

Primers used for other probes have been already described [5,29,31]
